# Exploring the mechanisms underlying the therapeutic effect of the *Radix Bupleuri-Rhizoma Cyperi* herb pair on hepatocellular carcinoma using multilevel data integration and molecular docking

**DOI:** 10.18632/aging.204388

**Published:** 2022-11-18

**Authors:** Luzhi Qing, Botao Pan, Yanjun He, Yu Liu, Minhong Zhao, Bo Niu, Xiuan Gao

**Affiliations:** 1 Affiliated Foshan Maternity and Child Healthcare Hospital, Southern Medical University, Foshan 528000, PR China; 2 Emergency Department, Affiliated Foshan Maternity and Child Healthcare Hospital, Southern Medical University, Foshan 528000, PR China

**Keywords:** traditional chinese medicine (TCM), *Radix Bupleuri-Rhizoma cyperi* herb pair (CXP), hepatocellular carcinoma (HCC), cell cycle, molecular docking

## Abstract

Traditional Chinese medicine (TCM) is a promising and effective treatment for cancer with minimal side effects through a multi-active ingredient multitarget network. *Radix Bupleuri* and *Rhizoma Cyperi* are listed as herbs dispersing stagnated liver Qi in China. They have been used clinically to treat liver diseases for many years and recent pharmacological studies have shown that they inhibit the proliferation of hepatocellular carcinoma (HCC). However, the pharmacological mechanisms, potential targets, and clinical value of the *Radix Bupleuri-Rhizoma Cyperi* herb pair (CXP) for suppressing HCC growth have not been fully elucidated. We identified 44 CXP targets involved in the treatment of HCC using the GEO dataset and HERB database. An analysis of the Traditional Chinese Medicine System Pharmacology Database (TCMSP) showed that CXP exerts synergistic effects through 4 active ingredients, including quercetin, stigmasterol, isorhamnetin, and kaempferol. GO and KEGG analyses revealed that CXP mainly regulates HCC progression through metabolic pathways, the p53 signaling pathway, and the cell cycle. Additionally, we applied The Cancer Genome Atlas (TCGA)-liver hepatocellular carcinoma (LIHC) database to perform the expression patterns, clinical features, and prognosis of 6 genes (CCNB1, CDK1, CDK4, MYC, CDKN2A, and CHEK1) in cell cycle pathways to reveal that CXP suppresses HCC clinical therapeutic value. Moreover, based on molecular docking, we further verified that CXP exerts its anti-HCC activity through the interaction of multiple active components with cell cycle-related genes. We systematically revealed the potential pharmacological mechanisms and targets of CXP in HCC using multilevel data integration and molecular docking strategies.

## INTRODUCTION

The liver is a key organ for glucose storage, detoxification and processing of exogenous substances, lipid and cholesterol homeostasis, metabolism, endocrine regulation of growth signals, and immune surveillance [1]. However, it is susceptible to environmental and genetic risk factors that increase the incidence of liver cancer, including oncogenic viral infection with hepatitis B virus (HBV) or hepatitis C virus (HCV), alcoholism, metabolic syndrome associated with obesity and diabetes [2]. Currently, hepatocellular carcinoma (HCC) is a global health problem with increasing morbidity and mortality rates. HCC accounts for approximately 90% of all primary liver cancers, is the most common type of liver cancer, and is the fourth leading cause of death worldwide [3, 4]. At present, four commonly used treatment methods are available for early-stage HCC, namely, transcatheter arterial chemoembolization, liver transplantation, radiofrequency ablation, and surgical resection [5, 6]. In fact, most patients are diagnosed with HCC in the middle or late stages of the disease, and thus only systemic therapies (targeted therapy and immunotherapy) are still available for these patients. Several first-line drugs, including sorafenib and lenvatinib, have been shown to improve patient overall survival when administered as single agents [7]. However, only approximately 30% of patients benefit from sorafenib, and the therapy has moderate or severe side effects [8]. Therefore, the development of more effective and less toxic HCC therapies is still urgently needed.

Traditional Chinese medicine (TCM), one of the most popular complementary and alternative medicine models in China, has been used clinically in Asia for thousands of years. Meanwhile, TCM has been gradually accepted by non-Chinese people because of its efficacy, accessibility, and lower toxicity. Several studies have shown that TCM formulas used alone or as an adjunct to conventional chemotherapy have shown good efficacy in the clinical treatment of cancer, including HCC and lung cancer [9–13]. In TCM theory, the synergistic effect of ‘herb-pair’ (Yaodui in Chinese) plays a key role in prescriptions [14]. Both *Radix Bupleuri* and *Rhizoma Cyperi* are considered the chief herbs for soothing the liver, and their combination is believed to produce a synergistic effect that increases the efficacy, but this synergistic effect still lacks support from modern pharmacological evidence. Nevertheless, *Radix Bupleuri* (Chai Hu)-*Rhizoma Cyperi* (Xiang Fu), a common herb pair, is widely used as an important ingredient in liver-soothing prescriptions in China. For example, *Radix Bupleuri* (Chai Hu)-*Rhizoma Cyperi* (Xiang Fu) herb pair (CXP) is an important component of the classic prescription Chaihu Shugan San (CSS), which was first recorded in the ‘Jing Yue Quan Shu’ in the Ming Dynasty [15]. Modern pharmacological studies have shown that CSS inhibits hepatic injury, especially in individuals with NAFLD [16, 17]. Furthermore, studies have shown that *Radix Bupleuri* or *Rhizoma Cyperi* administered as a single herb exhibits significant anticancer activity against HCC cells [18, 19]. Additionally, phytochemicals in both herbs have been reported to exert anticancer effects *in vivo* and *in vitro*, including on HCC [20, 21]. However, the comprehensive ingredients in CXP and the mechanisms underlying its multiple synergistic anti-HCC effects have not yet been completely elucidated.

**Figure 1:**
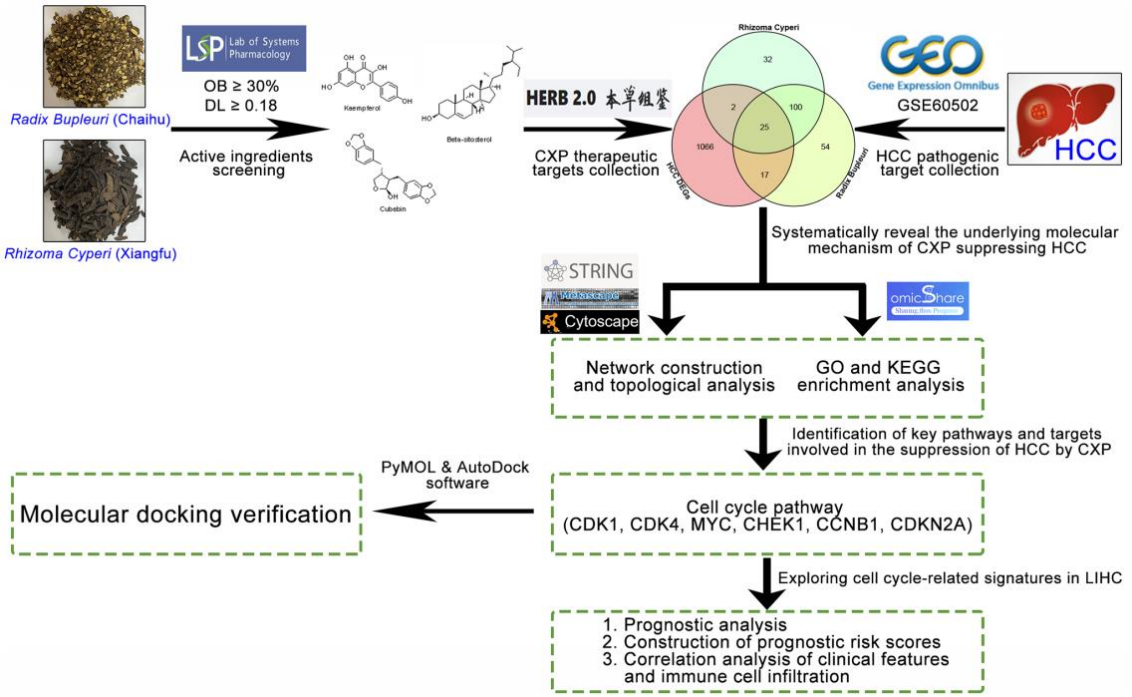
Flowchart of the analytical procedures used in the study.

Despite many technological advances, experimental elucidation of the interaction of a large number of compounds in CXP with proteins in HCC and the underlying pharmacological mechanisms is still difficult. With the rapid development of systems biology and polypharmacology, the emergence of network pharmacology has provided great opportunities for breakthroughs in TCM research [22]. To date, this method has been successfully used to elucidate the multitarget effects of TCM treatments on various diseases [23, 24]. It not only effectively bridges the gap between Western medicine and traditional medicine but also improves our understanding of the pharmacological mechanisms underlying the synergistic effects of herb pairs. In the present study, we employed various biological databases and biocomputational methods to investigate the pharmacological network of CXP in HCC and predict active compounds and their potential protein targets and pharmacological mechanisms. The overall flowchart of the study is shown in Figure 1.

## RESULTS

### Identification of significantly differentially expressed genes (DEGs) in HCC

**Figure 2:**
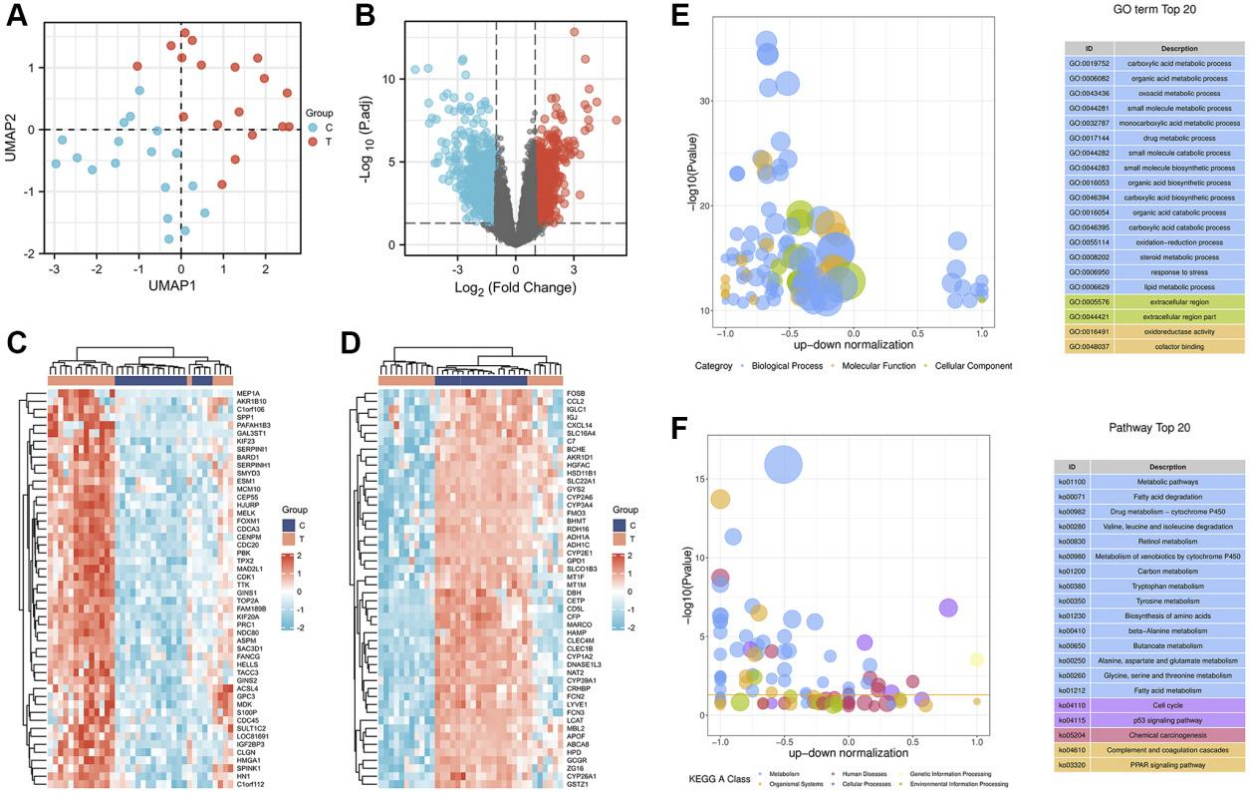
**Identification and enrichment analysis of DEGs in adjacent nontumor liver tissues and HCC tissues.** (**A**) The UMAP scatter plot. (**B**) The expression patterns of DEGs are shown in volcano plots. Red and blue dots represent upregulated genes (log2FC ≥ 1) and downregulated genes (log2FC ≤ −1), respectively, while gray represents genes with no significant difference in expression (P.adj < 0.05). Heatmap analysis of the top 50 up- (**C**) and downregulated (**D**) DEGs. Bubble plot showing the top 20 enriched GO terms (**E**) and KEGG (**F**) pathways. The larger the ordinate value in the bubble chart, the more significant the corresponding GO or KEGG result. The abscissa represents the normalized upregulation and downregulation value (the ratio of the difference between the number of upregulated genes and the number of downregulated genes to the total number of DEGs). The higher the value, the greater the number of upregulated genes enriched in the GO/KEGG pathway; conversely, the lower the value, the higher the number of downregulated genes enriched in the GO/KEGG pathway.

The GEO dataset GSE60502 was analyzed to identify DEGs in adjacent nontumor liver tissues and HCC tissues. First, an expression matrix was constructed with 18 pairs of samples, the nonlinear dimensionality reduction algorithm UMAP was used to determine two clusters, and each sample was assigned to the nearest cluster. The results showed a clear difference between the two groups (Figure 2A). Notably, 1110 DEGs (491 up-regulated and 619 down-regulated) were identified between noncancerous and cancerous tissues, as shown in the volcano plot (Figure 2B). In addition, subsets of the top 50 up- and downregulated DEGs were depicted in heatmaps (Figure 2C and 2D).

### Functional enrichment analysis of DEGs in HCC

A GO enrichment analysis was performed to further investigate the biological functions of the 1110 DEGs. We constructed bubble plots to display the top 20 enriched GO terms. The top 20 enriched GO terms showed that these DEGs were mainly enriched in metabolic processes (Figure 2E), including the following: “carboxylic acid metabolic process (GO:0019752)”, “organic acid metabolic process (GO:0006082)”, “oxoacid metabolic process (GO:0043436)”, “small molecule metabolic process (GO:0044281)”, “monocarboxylic acid metabolic process (GO:0032787)”, “drug metabolic process (GO:0017144)”, “steroid metabolic process (GO:0008202)”, and “lipid metabolic process (GO:0006629)”. Moreover, these metabolic processes were mainly regulated by the downregulation of DEGs.

Additionally, in the KEGG pathway analysis, the top 20 KEGG pathways related to the 1110 DEGs were identified (Figure 2F). KEGG pathways were particularly enriched in three categories, including metabolism, organismal systems, and cellular processes. In particular, 15 of the 20 pathways were involved in the metabolism category, including “Metabolic pathways (ko01100)”, “Fatty acid degradation (ko00071)”, “Drug metabolism – cytochrome P450 (ko00982)”, “Metabolism of xenobiotics by cytochrome P450 (ko00980)”, “Fatty acid metabolism (ko01212)”, Tyrosine metabolism (ko00350)”, and “Carbon metabolism (ko01200)”. The category of organismal systems consists of two pathways, “Complement and coagulation cascades (ko04610)” and “PPAR signaling pathway (ko03320)”. The cellular processes category also consists of two pathways, “Cell cycle (ko04110)” and “p53 signaling pathway (ko04115)”. Notably, among these 20 KEGGs, upregulated DEGs were only significantly enriched in the cellular process category.

### Protein–protein interaction (PPI) network and module analysis of DEGs in HCC

**Figure 3:**
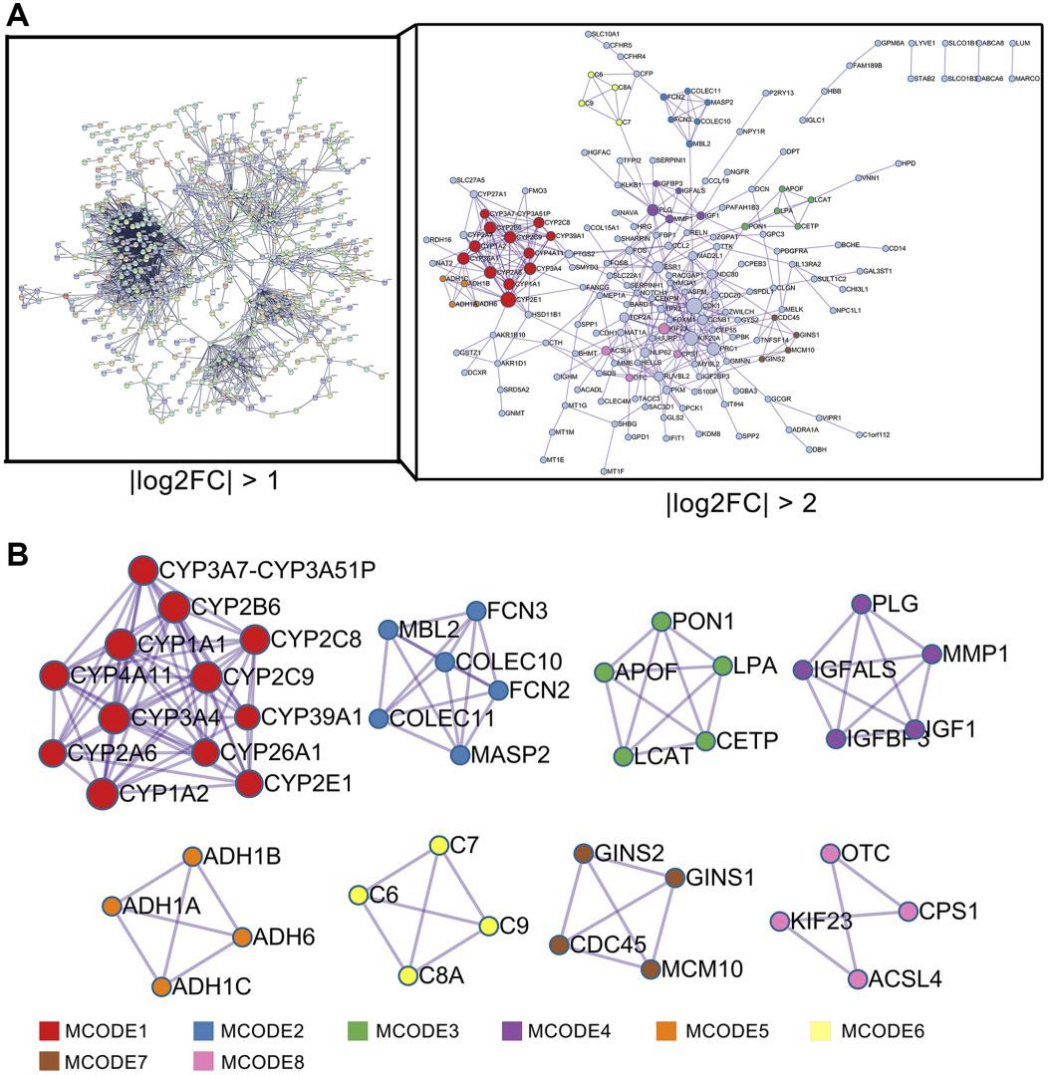
**PPI network analysis of DEGs in HCC.** (**A**) PPI network analysis of 1110 DEGs (right panel, STRING database) and 224 DEGs (left panel, Metascape web tool). (**B**) MCODE module for the gene clustering analysis.

Next, the PPI network was constructed to reveal the interconnections and roles of these 1110 DEGs. These 1110 DEGs (|log2FC| >1) were input into the STRING database to construct a PPI network. The results were obtained according to an interaction score ≥0.9 and hidden disconnected nodes to improve the reliability of the PPI network [25]. This PPI network is extremely complex, containing 1080 nodes and 2405 interactions (Figure 3A). By calculating the degree of this PPI network, we concluded that CDK1 (degree = 91), CCNB1 (degree = 72), and TOP2A (degree = 62) were the top three hub genes in this network (Supplementary Table 1). Limited by the complexity of the aforementioned network, we next selected DEGs with |log2FC| >2 to further construct the PPI network and deeply explored the key connections and hub genes of the network. A total of 224 DEGs (71 up- and 153 downregulated genes) were screened and input into the Metascape web tool to construct a PPI network consisting of 171 nodes and 404 edges. CDK1 (degree = 27), CYP2E1 (degree = 19), and KIF20A (degree = 17) were hub genes with higher node degrees in this PPI network (Supplementary Table 2). The Molecular Complex Detection (MCODE) algorithm was then applied to this network to identify neighborhoods where proteins were densely connected. Eight MCODE networks identified for individual gene lists have been gathered and are shown in Figure 3B. Furthermore, pathway and process enrichment analyses were independently applied to each MCODE component, and the three best-scoring terms based on *P* values were retained as functional descriptions of the corresponding components, as shown in Supplementary Table 3. Most of these MCODE components were enriched in metabolism-related pathways or processes.

### Active compounds and potential therapeutic targets of CXP

**Figure 4:**
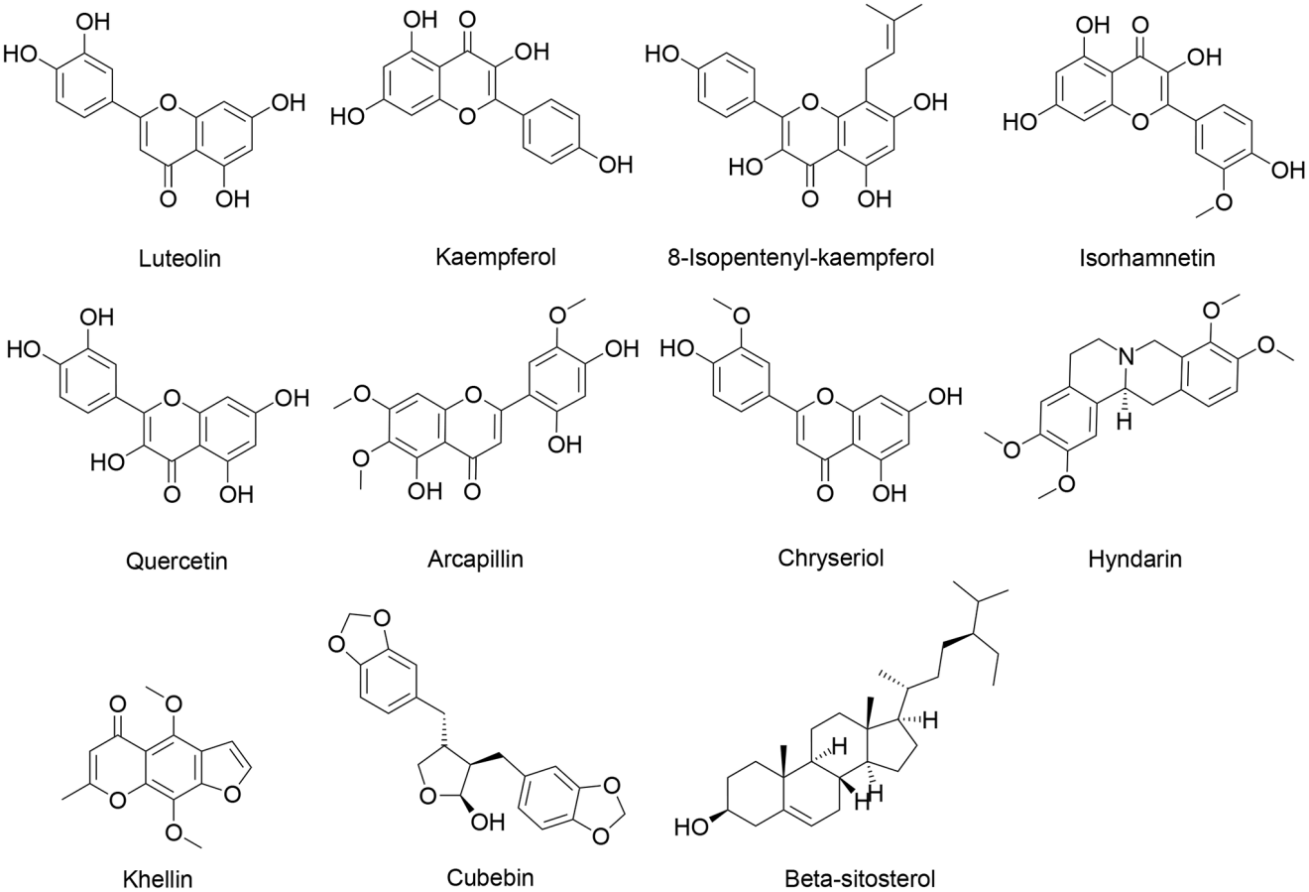
Chemical structures of some active ingredients of CXP.

According to the two criteria of drug-likeness (DL) ≥0.18 and oral bioavailability (OB) ≥30% [26, 27], 31 CXP-active compounds were identified in the Traditional Chinese Medicine System Pharmacology Database (TCMSP) (Supplementary Table 4). The results showed that *Radix Bupleuri* and *Rhizoma Cyperi* contained 17 and 18 active ingredients, respectively, and both contained the four active compounds quercetin, stigmasterol, isorhamnetin, and kaempferol. The structures of these compounds are shown in Figure 4. After removing duplicate values, 196 potential targets of 17 active compounds of *Radix Bupleuri* were obtained from the HERB database. Similarly, 159 potential targets of 18 active compounds of *Rhizoma Cyperi* were obtained. Finally, 230 potential targets of 31 CXP active ingredients were identified (Supplementary Table 5).

### Target screening and network analysis of CXP in the treatment of HCC

**Figure 5:**
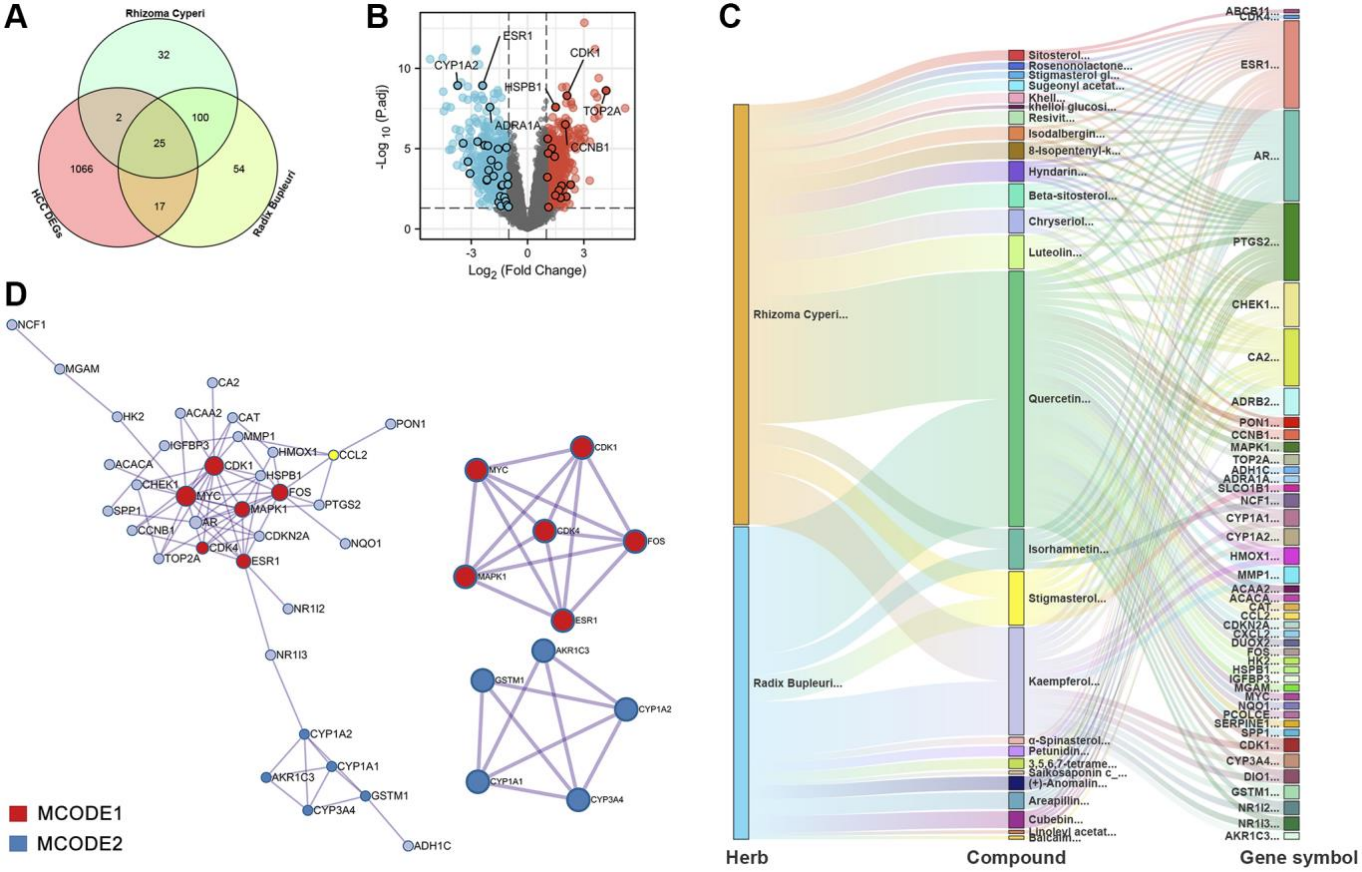
**PPI and H-C-T network analysis of 44 potential therapeutic targets for CXP in HCC.** (**A**) Venn diagram. (**B**) The distribution of 44 potential therapeutic targets of CXP in the treatment of HCC in the volcano plot of DEGs in HCC. (**C**) H-C-T network analysis. (**D**) PPI network and gene clustering analysis (Metascape web tool).

The comparison of the potential targets of the active components in CXP with the 1110 candidate targets of the DEGs from the mRNA array in HCC showed an overlap of 44 targets, as shown in Figure 5A and Supplementary Table 5. The specific positions of these 44 targets in the HCC-DEGs volcano plot are shown in Figure 5B, and 16 targets were upregulated and 28 were downregulated in HCC. Among them, CDK1, TOP2A, CCNB1, ESR1, CYP1A2, and ADRA1A had higher -Log10 (P.adjust) in the volcano plot of HCC-DEGs. In the Venn diagram, both *Radix Bupleuri* and *Rhizoma Cyperi* exerted anti-HCC effects through 25 common potential targets. We speculated that these comment targets may explain why CXP exerts a synergistic anti-HCC pharmacological effect. We constructed a network of Herb-Compound-Targets (H-C-T) to further understand the interconnectedness of herbs, active compounds, and potential anti-HCC targets (Figure 5C). Both herbs exerted their anti-HCC effects on multiple targets mainly through four active ingredients, namely, quercetin, stigmasterol, isorhamnetin, and kaempferol. Meanwhile, the results suggested that ESR1, AR, PTGS2, CHEK1, and CA2 are the main targets regulated by multiple active components of CXP.

We constructed a PPI network and performed MCODE analysis and annotation on this network to further investigate the intrinsic connectivity of potential anti-HCC targets of CXP (Figure 5D). The PPI network contained 73 connections and 2 MCODE networks. Notably, MCODE2 annotated 3 metabolic pathways or processes with significant enrichment, including “estrogen metabolism (log10 (P) = −12.3)”, “long-chain fatty acid metabolic process (log10 (P) = −12.2)”, and “fatty acid biosynthetic process (log10 (P) = −12.1)” (Supplementary Table 6). All mapped intersecting proteins were input into Cytoscape software to calculate the topological parameters of the PPI network related to CXP against HCC. The analysis identified that the genes in the MCODE1 and MCODE2 networks had high degrees, including the proteins CDK1 and CDK4, which are related to the regulation of the cell cycle, and the proteins CYP1A2, GSTM1, CYP3A4, and CYP1A1, which are related to metabolism (Supplementary Table 7).

### Functional enrichment and network analyses of potential targets of CXP for the treatment of HCC

**Figure 6:**
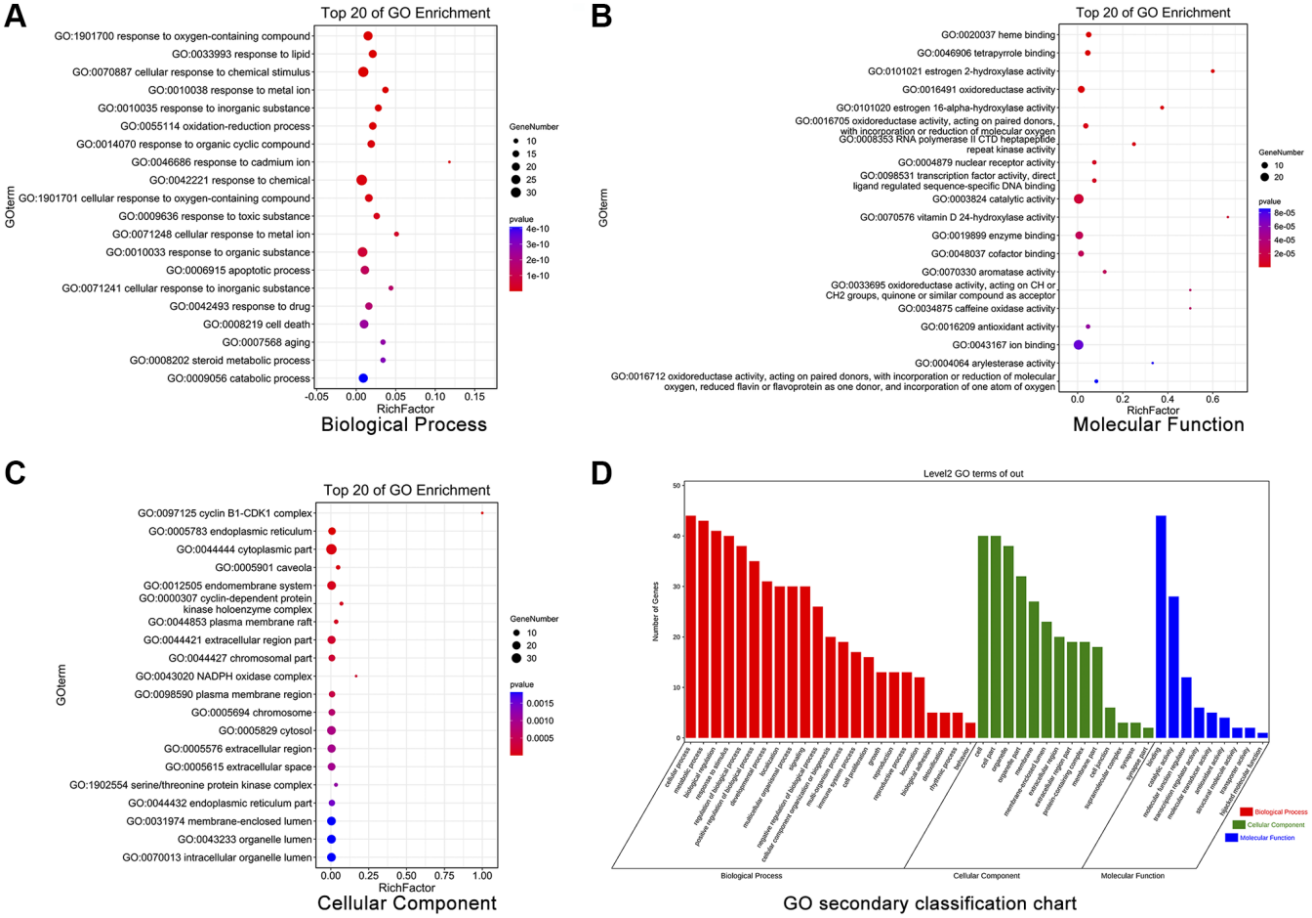
**GO enrichment analysis of 44 potential therapeutic targets for CXP in HCC.** (**A**) Biological processes. (**B**) Molecular functions. (**C**) Cellular components. (**D**) Secondary classification chart of enriched GO terms.

The GO enrichment analysis identified the top 20 GO terms in the cellular component, molecular function, and biological process categories. In terms of these top 20 GO terms (Figure 6A–6C), CXP treatment of HCC mainly involves the regulation of “response to oxygen-containing compound (GO:1901700)”, “response to lipid (GO:0033993)”, “cellular response to chemical stimulus (GO:0070887)”, “heme binding (GO:0020037)”, “tetrapyrrole binding (GO:0046906)”, “estrogen 2-hydroxylase activity (GO:0101021)”, “cyclin B1-CDK1 complex (GO:0097125)”, “endoplasmic reticulum (GO:0005783)”, and “cytoplasmic part (GO:0044444)”, among others. Moreover, we performed a secondary classification of all the enriched GO terms, and the results showed that in the biological process category, the GO terms were mainly involved in cellular processes, metabolic processes, and biological regulation (Figure 6D).

**Figure 7:**
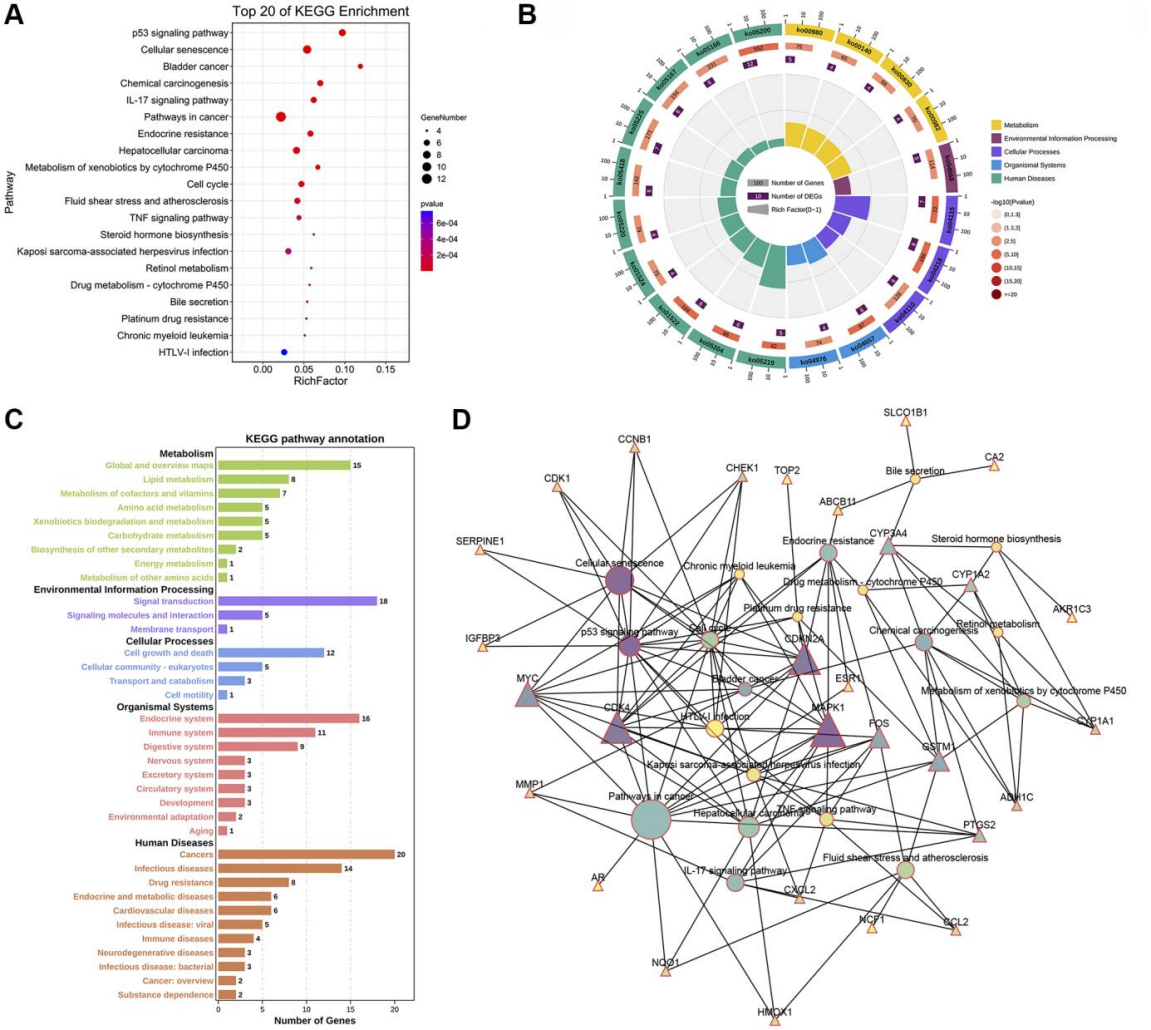
**KEGG enrichment and KEGG pathway-gene network analyses of 44 potential therapeutic targets for CXP in HCC.** (**A**) Top 20 KEGG pathways. (**B**) Secondary classification of the top 20 KEGG pathways. (**C**) Secondary classification of all KEGG pathways. (**D**) KEGG pathway-gene network.

In addition, we identified the top 20 KEGG pathways involved in CXP treatment of HCC by performing a KEGG enrichment analysis, including “p53 signaling pathway (ko04115)”, “Cellular senescence (ko04218)”, “Bladder cancer (ko05219)”, “Chemical carcinogenesis (ko05204)”, “IL-17 signaling pathway (ko04657)”, “Pathways in cancer (ko05200)”, “Endocrine resistance (ko01522)”, “Hepatocellular carcinoma (ko05225)”, “Metabolism of xenobiotics by cytochrome P450 (ko00980)”, “Cell cycle (ko04110)”, “Fluid shear stress and atherosclerosis (ko05418)”, “TNF signaling pathway (ko04668)”, “Steroid hormone biosynthesis (ko00140)”, “Kaposi sarcoma-associated herpesvirus infection (ko05167)”, “Retinol metabolism (ko00830)”, “Drug metabolism-cytochrome P450 (ko00982)”, “Bile secretion (ko04976)”, “Platinum drug resistance (ko01524)”, “Chronic myeloid leukemia (ko05220)”, and “HLCV-I infection (ko05166)” (Figure 7A). Six of these top 20 KEGG pathways coincided with the previous top 20 KEGG pathways of DEGs-HCC. Next, we conducted a secondary classification of the top 20 KEGG pathways, and these KEGG pathways were mainly divided into 5 categories, including metabolism (4), environmental information processing (1), cellular processes (3), organismal systems (2), and human diseases (10) (Figure 7B). Likewise, we performed secondary classification of all KEGG pathways, and the results are shown in Figure 7C. In the category of metabolism, the KEGG pathways were mainly enriched in lipid metabolism, amino acid metabolism, xenobiotics biodegradation and metabolism. In cellular processes, it is mainly enriched in cell growth and death, cellular community-eukaryotes, and transport and catabolism. We constructed a KEGG pathway-gene (K-G) network to further mine the hub genes involved in the top 20 KEGG pathways, and the results showed that MAPK1, CDKN2A, CDK4, MYC, and GSTM1 had greater degrees (Figure 7D).

### Identification of six cell cycle-related genes involved in the effects of CXP on HCC and a prognostic analysis

**Figure 8:**
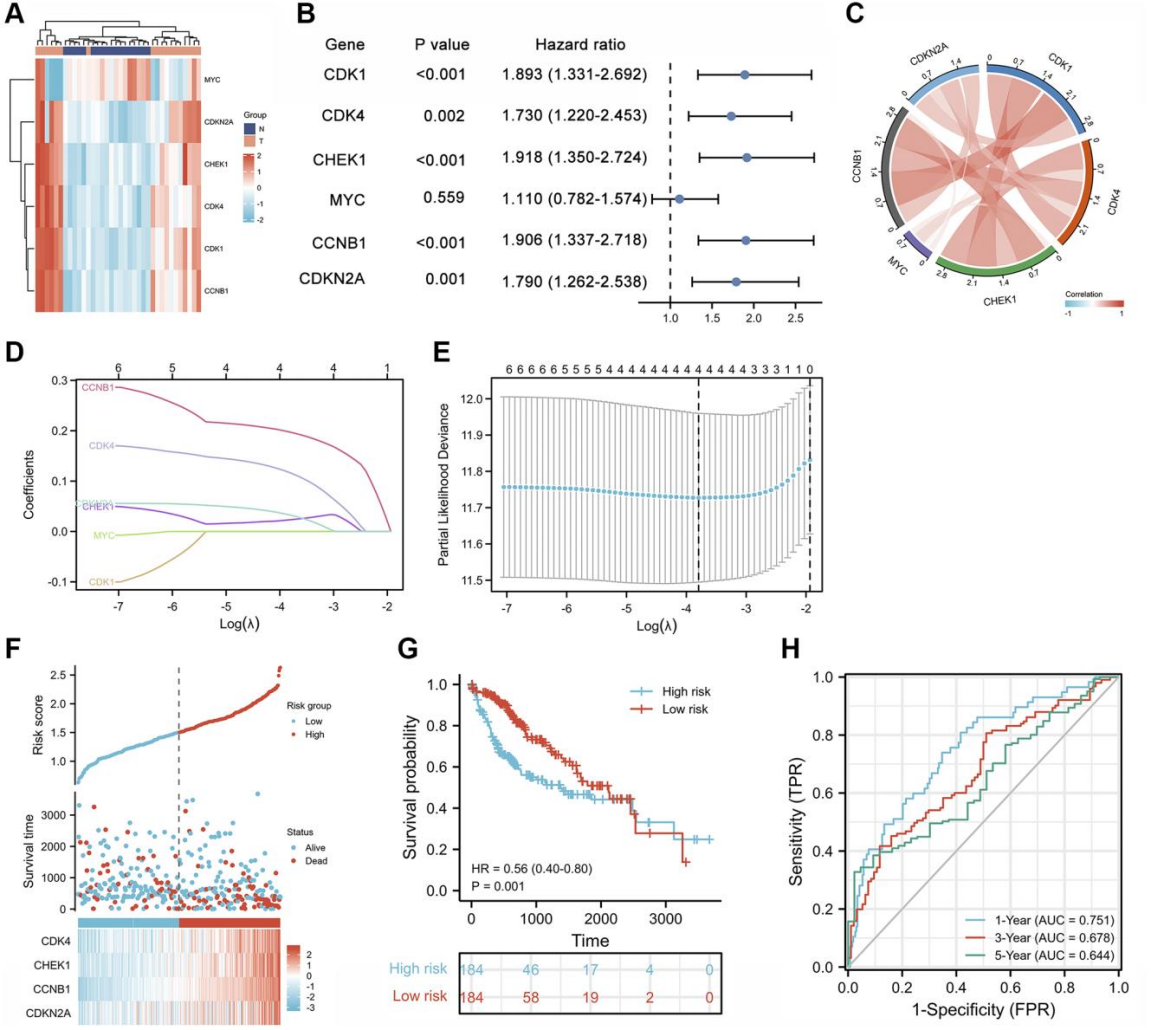
**Prognostic analysis of cell cycle-related genes and establishment of a prognostic model.** (**A**) Heatmap of the expression patterns of 6 cell cycle-related genes in 18 pairs of adjacent non-tumor liver tissues and HCC tissues. (**B**) Forest plot of the univariate Cox analysis of 6 cell cycle-related genes. (**C**) Correlation network of 6 cell cycle-related genes. (**D**) LASSO coefficient profiles of 6 cell cycle-related genes. (**E**) Cross-validation for tuning parameter selection in the LASSO regression analysis. (**F**) The distribution of risk scores, gene expression levels, and survival status of patients with LIHC in the training cohort. (**G**) Kaplan–Meier curves of the OS of all patients with LIHC in TCGA cohort based on the risk score. (**H**) Time-dependent ROC curve analysis of the prognostic model (1, 3, and 5 years).

Combined with the aforementioned analysis of hub genes and pathways, we speculated that the cell cycle pathway plays a key role in CXP treatment of HCC. CXP regulates this pathway to suppress HCC via six genes, including CDK1, CDK4, CCNB1, CHEK1, CDKN2A, and MYC. The heatmap was used to show the expression patterns of these 6 cell cycle-related genes in 18 pairs of adjacent non-tumor liver tissues and HCC tissues (Figure 8A). The results showed that except for MYC, the transcript levels of the other 5 genes were abnormally high in HCC. Univariate Cox proportional hazards regression analysis found that five cell cycle-related genes were associated with the prognosis of HCC, and CDK1, CDK4, CHEK1, CCNB1, and CDKN2A were considered risk factors (*P* < 0.01, HR >1) (Figure 8B). In addition, except MYC, five prognostic cell cycle-related genes were strongly positively correlated with each other (Figure 8C).

### Construction of prognostic risk scores with cell cycle-related genes in TCGA dataset

The five cell cycle-related genes (CDK1, CDK4, CCNB1, CHEK1, and CDKN2A) were analyzed using the least absolute shrinkage and selection operator (LASSO) Cox regression analysis to construct a cell cycle-related signature for predicting survival. Four cell cycle-related genes (CDK4, CHEK1, CCNB1, and CDKN2A) were used to establish a risk score to predict the overall survival (OS) of patients with hepatocellular carcinoma (LIHC) in TCGA training set using LASSO Cox regression analysis (Figure 8D and 8E). We established a risk score formula based on the expression of four genes in patients with LIHC and then divided patients from TCGA training set into low-risk and high-risk groups based on the median risk score. The distribution of the cell cycle-related signature score, the survival status, and a heatmap exhibiting the expression profiles of the 4 genes in the high- and low-risk groups are presented in Figure 8F. Meanwhile, the Kaplan-Meier survival analysis showed that patients in the low-risk group had significantly longer OS times than those in the high-risk group (Figure 8G, HR (95% CI) = 0.56 (0.40–0.80), *P* = 0.001). Subsequently, a time-dependent receiver operating characteristic (ROC) analysis was performed, and the results showed that the risk score performed well in predicting 1-, 3-, and 5-year OS, with areas under the curves (AUCs) of 0.751, 0.678, and 0.644, respectively (Figure 8H).

### Correlation analysis of clinical characteristics and immune cell infiltration based on the expression of four cell cycle-related genes in LIHC

**Figure 9:**
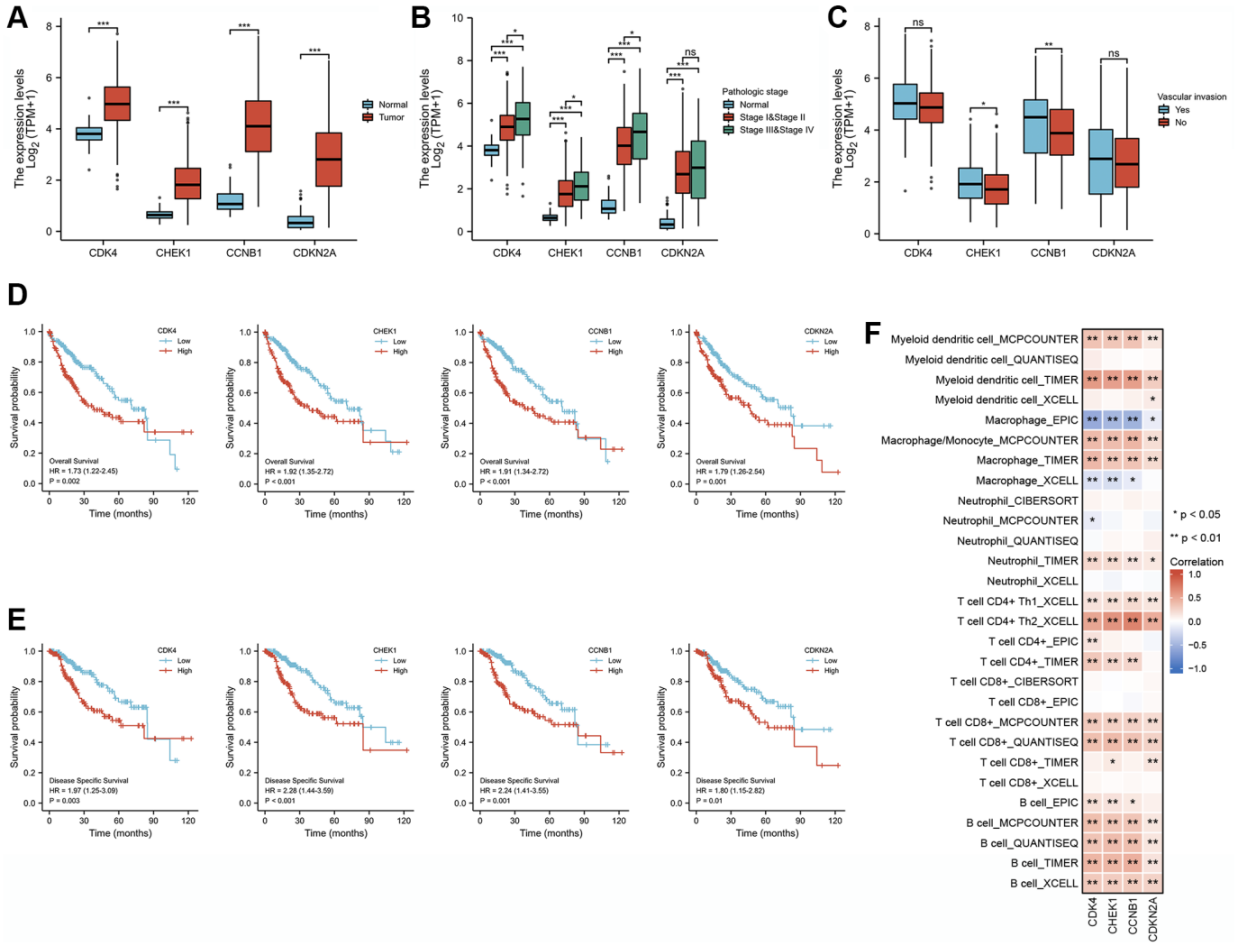
**Correlation analysis of the expression of four cell cycle-related genes with clinical features and immune cell infiltration in patients with LIHC.** (**A**) The differential expression of CCNB1, CDK4, CDKN2A, and CHEK1 between normal and tumor tissues. (**B**) CCNB1, CDK4, CDKN2A, and CHEK1 mRNA expression in normal individuals or individuals with different pathologic stages (stage I and II, and stage III and IV). (**C**) Differences expression of CCNB1, CDK4, CDKN2A, and CHEK1 mRNA in patients with different types of vascular invasion. (**D**) Kaplan-Meier curves of OS for different cell cycle-related genes. (**E**) Kaplan-Meier curves of DSS for different cell cycle-related genes. (**F**) Correlation analysis between four cell cycle-related genes and infiltration levels of different immune cells estimated using TIMER, EPIC, XCELL, CIBERSORT, and QUANTISEQ. ^*^, ^**^, and ^***^ represent *P* < 0.05, *P* < 0.01, and *P* < 0.001, respectively.

We analyzed the clinical characteristics of the 4 cell cycle-related genes involved in CXP treatment of HCC, including CDK4, CHEK1, CCNB1, and CDKN2A, to further evaluate the clinical application value of CXP in HCC treatment. We assessed RNA-seq data from 374 HCC tissues and 50 adjacent normal tissues using transcriptome data from TCGA-LIHC database to investigate the roles of these genes in LIHC. The expression of all four genes was elevated in tumor tissues compared with normal liver tissues (*P* < 0.05) (Figure 9A). We then explored the expression patterns of these four cell cycle-related genes across vascular invasion subtypes and pathologic stages in these samples to identify the causes of disease diversification and specific clinical outcomes. The expression of these genes was increased in stage III and IV compared to stage I and II (*P* < 0.05) (Figure 9B); meanwhile, the expression of the CCNB1 and CHEK1 genes was significantly higher in HCC tissues with vascular invasion than in HCC tissues without vascular invasion (*P* < 0.05) (Figure 9C). Moreover, we further tested the clinical efficacy of these genes in LIHC. Analysis of TCGA-LIHC dataset revealed that patients with LIHC presenting high expression levels of these genes had unfavorable overall OS and disease specific survival (DSS) (*P* < 0.05) (Figure 9D and 9E).

We next analyzed the correlation between the abundance of 6 immune cell markers and the expression of 4 cell cycle-related genes using RNA-seq data and the TIMER2.0 database. The correlation analysis showed that the levels of infiltrating B cells, CD8+ T cells, CD4+ T cells, macrophages, and myeloid dendritic cells were positively correlated with the expression level of 4 the cell cycle-related genes (CDK4, CHEK1, CCNB1, and CDKN2A) in LIHC (Figure 9F, *P* < 0.05). These results speculate that the expression of the four cell cycle-related genes in LIHC was related to different degrees of immune cell infiltration through different pathways, further supporting that these four cell cycle-related genes may be effective factors affecting patient survival and prognosis.

### Component-target docking analysis

**Figure 10:**
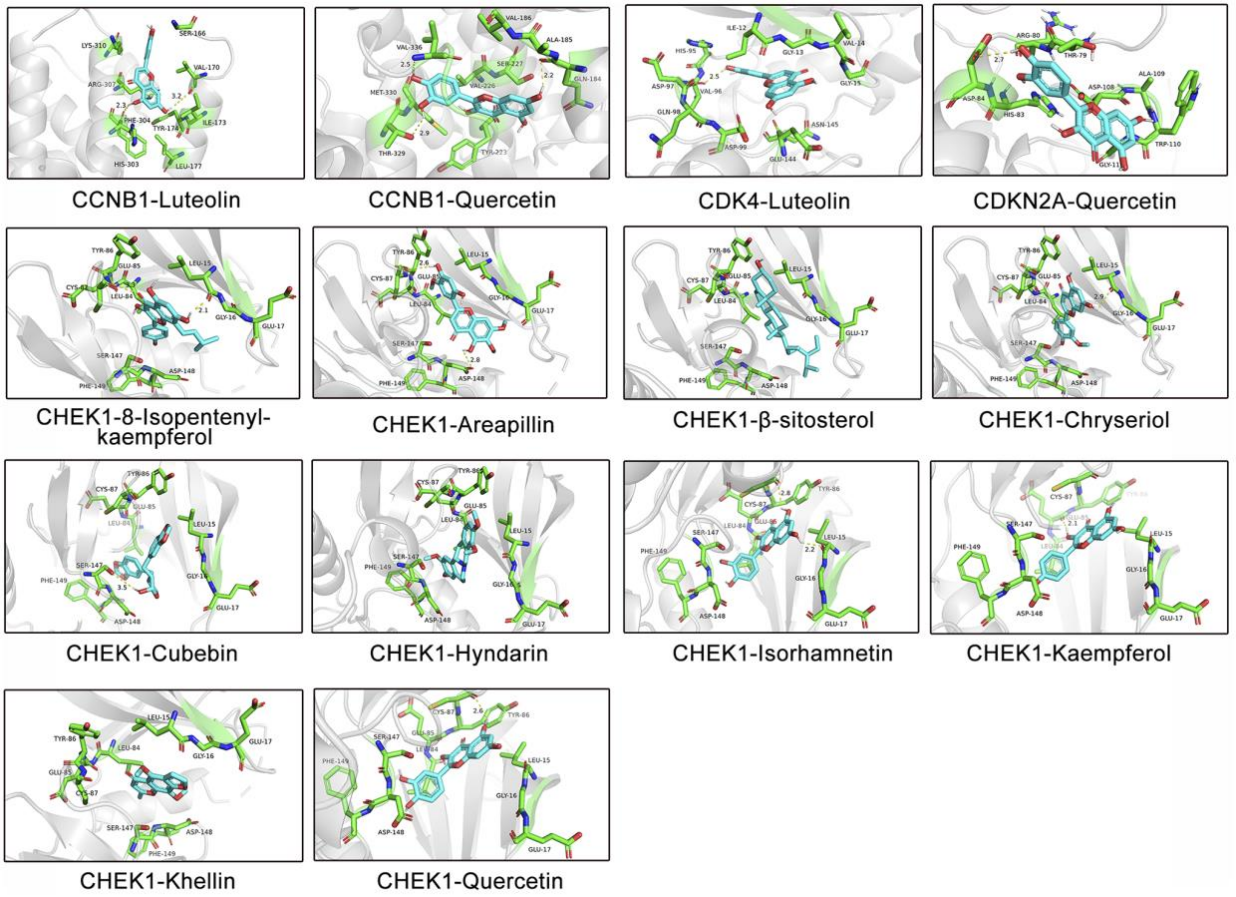
**Molecular models of the binding of different active ingredients to 4 cell cycle-related targets, which are shown as predicted protein–ligand binding diagrams and 3D interaction diagrams displayed using PyMOL.** Green represents the surrounding amino acid residues in the binding pocket, and cyan represents the active ingredient.

The aforementioned results suggest that CXP suppresses HCC growth through multiple active components acting on multiple hub targets. As an approach to improve the reliability of the results, we used a molecular docking strategy to simulate the binding mode between the active components of CXP and the targets, and we calculated the binding energy to explain the mechanism by which CXP inhibits HCC growth. According to the predicted results from molecular docking, multiple active components (luteolin, quercetin, kaempferol, 8-isopentenyl-kaempferol, arcapillin, β-sitosterol, chryseriol, cubebin, hyndarin, isorhamnetin, and khellin) bind these cell cycle-related targets (CCNB1, CDK4, CDKN2A, and CHEK1) well with low binding energy (Supplementary Table 8 and Figure 4). This efficient binding may be because these active ingredients contain multiple hydroxyl groups, making them good hydrogen bond donors or acceptors. As shown in Figure 10, the docking results predict that these active ingredients form stable non-covalent interactions with these selected proteins. An analysis of the way proteins interact with ligands may lead to the conclusion that these active components bind well to these selected targets, mainly by forming multiple hydrogen bonds to obtain lower binding energies. Furthermore, molecular docking models provide evidence for how CXP acts on these targets to suppress HCC growth.

## DISCUSSION

The pathological mechanism of HCC is very complex and involves multiple targets and signaling pathways during its progression. We used the public GEO dataset to analyze the gene expression profiles of human liver cancer tissues relative to adjacent tissues as a method to understand the genes and mechanisms that regulate HCC progression. A total of 1110 DEGs were identified, including 491 upregulated genes and 619 downregulated genes, indicating that the progression of HCC is regulated by a complex and large gene network. Most of these DEGs are involved in regulating cellular metabolism or cellular processes. Published studies have shown that DEGs, including MEP1A [28], AKR1B10 [29], CDK1 [30], and ADH1A [31], are involved in HCC growth. The liver is the main site of biotransformation, and its abnormal metabolism significantly alters the progression of liver cancer. Metabolic alterations clearly characterize HCC tumors. Our KEGG enrichment analysis indicated that multiple metabolic pathways are involved in the progression of HCC, consistent with published studies [32]. Moreover, cellular process-related pathways, including the cell cycle and p53 pathways, are also involved in the pathology of HCC. A study by Zhu et al. [33] confirmed that p53 deficiency affects cholesterol esterification and exacerbates hepatocarcinogenesis. Song et al. [34] showed that reticulon 3-mediated activation of Chk2/p53 inhibit hepatocellular carcinogenesis. Moreover, some existing targeted therapies exert anti-HCC effects through specific signals, including anti-angiogenesis or cell cycle progression [35]. These results suggest that the pathological mechanism of HCC is very complex and involves the regulation of multiple genes and signaling pathways during progression. This finding necessitates the development of a therapeutic strategy that modulates multiple targets in HCC to improve patient outcomes.

TCM, as a multicomponent and multitarget empirical therapy, has been used clinically in Asia for thousands of years. Because of this unique feature, TCM treatments are considered promising therapeutic strategies for complex diseases, including liver cancer [36]. *Radix Bupleuri* and *Rhizoma Cyperi*, herbs dispersing stagnated liver Qi, have been suggested to possess hepatoprotective activity in modern pharmacological studies [20, 37, 38]. Su et al. [39] showed that a *Radix Bupleuri* water extract reduced the viability of HepG2 cells and that *Radix Bupleuri* enhanced the pharmacological effects of 5-fluorouracil-induced HepG2 cell death. Furthermore, as shown in the study by Mannarreddy et al. [19], the methanol extract of *Rhizoma Cyperi* displays significant anticancer activity against multiple cancer cell lines, including HepG2, without inhibiting noncancer cells. However, the pharmacological mechanisms involved in the suppressing HCC remain largely unknown. In addition, *Radix Bupleuri* and *Rhizoma Cyperi* are often used as compatible drug pairs in in classic TCM prescriptions, such as CSS [16]. Therefore, studies exploring the molecular mechanism of the *Radix Bupleuri-Rhizoma Cyperi* drug pair in treating HCC are necessary.

However, due to the limitation of the complex molecular network characteristics of TCM in treating diseases, modern pharmacological research on TCM has not achieved great progress, which undoubtedly widens the research gap between TCM and Western medicine. In recent years, the introduction of network pharmacology has provided a good research strategy for investigating the modern pharmacology of TCM. Therefore, we attempted to utilize this strategy to explore how CXP exerts its pharmacological anti-HCC effects.

According to the TCMSP database, 17 and 18 key active ingredients were identified in *Radix Bupleuri* and *Rhizoma Cyperi*, respectively. These key active ingredients were mainly sterols (stigmasterol, sitosterol, β-sitosterol, etc.) and flavonoids (quercetin, luteolin, isorhamnetin, kaempferol, etc.). Notably, both herbs contain the following four active ingredients: quercetin, stigmasterol, isorhamnetin, and kaempferol. We hypothesized that these 4 active ingredients may be the important basis for the synergistic anti-HCC effect of the two herbs on CXP. Pan et al. [23] showed that the natural product quercetin derived from TCM inhibits the proliferation of HCC cell lines (HepG2 and Huh-7) by inhibiting the PI3K/AKT signaling pathway. Kin et al. [40] showed that stigmasterol induces apoptosis in human hepatoma HepG2 cells. Isorhamnetin, a natural antioxidant with significant cardioprotective effects, has been shown to enhance the anticancer activity of doxorubicin in HepG2 cells and has been used as an adjuvant therapy during the long-term clinical use of doxorubicin [41]. According to Han et al. [42], kaempferol induces autophagic cell death in various human hepatoma cells by activating AMPK signaling. In addition, other active ingredients have great potential in cancer therapy. For example, the naturally occurring furan chromone kellin has been used as an anticancer therapeutic agent [43], and the bioflavonoid troxerutin inhibits hepatic tumorigenesis by disrupting the MDM2-p53 interaction [44]. Overall, the anticancer pharmacological effects of these active ingredients may serve as an important theoretical basis for the clinical treatment of hepatic tumorigenesis using CXP.

Forty-four potential therapeutic targets of CXP against HCC were identified from the GEO dataset and the TCM target prediction database HERB. Subsequently, according to the H-C-T network diagram, CXP may exert its anti-HCC effect through the actions of multiple active components on multiple targets. Among them, the two herbs in CXP exert synergistic anti-HCC effects on multiple targets mainly through their shared active ingredients: quercetin, stigmasterol, isorhamnetin, and kaempferol. TCM prescriptions are a rational combination of a variety of herbal medicines based on TCM theoretical compatibility, but they still lack the support of modern pharmacology, which limits TCMs progress [45]. Here, we elucidated the rationale for the compatibility of CXP through network pharmacology strategies. Furthermore, the PPI results suggest that CXP inhibits HCC through a complex molecular network. Some DEGs with high topological parameters were defined as hub genes, including the oncogene MYC, which can drive hepatocarcinogenesis [46], the therapeutic target CDK4 for targeted therapy of HCC [47], and the metabolic enzyme CYP3A4, which is involved in the abnormal metabolism of HCC [48].

We performed enrichment analyses of 44 potential therapeutic targets to better elucidate the molecular mechanism of CXP against HCC. The results showed that CXP mainly regulates HCC progression through metabolic and cellular processes, including the p53 signaling pathway, cellular senescence, the cell cycle, and retinol metabolism. These enrichment results largely overlapped with our previous KEGG enrichment results for HCC progression, such as the involvement of the p53 signaling pathway and cell cycle. Moreover, the foregoing discussion has clarified that the progression of HCC is closely related to the p53 pathway and the cell cycle. Additionally, several cell cycle-related genes have recently been identified as potential therapeutic targets for the treatment of HCC, including CDK1 [49], CDK4 [47], CCNB1 [50], and CHEK1 [51]. In this study, we identified that CXP mediates HCC progression by acting on six genes regulating cell cycle pathways, including CCNB1, CDK1, CDK4, CDKN2A, MYC, and CHEK1. Therefore, we speculate that the molecular mechanism by which CXP suppresses HCC may be mediated by multiple active components acting on six genes regulating cell cycle pathways, including CCNB1, CDK1, CDK4, CDKN2A, MYC, and CHEK1. Interestingly, the molecular mechanism of CXP treatment of HCC involves cellular senescence, and TCM-induced cellular senescence has been recognized as a promising strategy for cancer treatment in recent years [52]. Cellular senescence is a process that produces anticancer activity by inducing irreversible cell cycle arrest and inhibiting the proliferative capacity of cells [53]. Therefore, we hypothesized that CXP may induce cellular senescence by acting on six cell cycle-related genes and inducing cell cycle arrest, thereby inhibiting the progression of HCC.

As the roles of cell cycle-related signatures in HCC are largely underexplored, a broader understanding of their expression profiles in cancer and prognostic analyses are important. In this study, we analyzed the expression patterns, prognostic value, and clinical characteristics of cell cycle-related genes in LIHC to determine the value of CXP clinical application in the treatment of HCC. We identified five cell cycle-related genes, CDK1, CDK4, CHEK1, CCNB1, and CDKN2A, which were significantly associated with the OS of patients with LIHC and were considered risk factors. Subsequently, the results of LASSO Cox regression model and analysis of the Kaplan-Meier survival curve revealed that patients with LIHC presenting low expression of cell cycle-related genes (CDK4, CHEK1, CCNB1, and CDKN2A) had a better prognosis than those with high expression. Moreover, the clinical characteristics of these genes were analyzed in the LIHC dataset, and the results revealed that their expression levels were closely related to the pathological stage of LIHC and the progression of vascular invasion. These data strongly suggest that CDK4, CHEK1, CCNB1, and CDKN2A are potential prognostic biomarkers for HCC and key therapeutic targets for CXP to suppress HCC.

Given the achievements of immunotherapy in the field of cancer treatment [54], revealing the link between cell cycle-related genes and the immune environment will help us better understand the molecular mechanism by which CXP inhibits HCC. The expression of CDK4, CCNB1, CHEK1, and CDKN2A correlated with the infiltration levels of various immune cells in HCC. The correlation between the expression of cell cycle-related genes and immune cells suggested roles for CDK4, CCNB1, CHEK1 and CDKN2A in regulating HCC tumor immunology. Despite the lack of in-depth data, these findings provide evidence for the future combination of CXP and immunotherapy to improve the prognosis of patients with HCC. Furthermore, we applied a molecular docking approach to illustrate that CXP modulates HCC through the effects of various active components on these cell cycle-related genes.

## CONCLUSIONS

In conclusion, we elucidate the underlying pharmacological mechanisms and targets of CXP action in HCC therapy by integrating multiple databases. CXP inhibits tumors through multiple metabolic pathways and cellular processes. An analysis of TCGA data showed that CXP may improve the prognosis and clinical outcomes of patients with HCC by regulating cell cycle-related genes, revealing its clinical application value. Furthermore, CDK4, CDKN2A, CCNB1, and CHEK1 genes were identified as key therapeutic targets for CXP treatment of HCC. In recent years, TCM has become a promising cancer treatment strategy because of its multiple active components and multiple targets. However, due to the complexity of TCM pharmacological mechanisms, TCM research has not been rapidly established, and the proposal of network pharmacology strategies is gradually changing this dilemma. To our knowledge, this study represents the first systematic pharmacological analysis of CXP-mediated HCC therapy. Therefore, although this study still has limitations, it provides innovative research methods and breakthroughs for TCM research.

## MATERIALS AND METHODS

### Data preparation

#### Chemical components in CXP

We collected the chemical components of CXP from the TCMSP (https://old.tcmsp-e.com/tcmsp.php), a database that provides the relationship between chemical components, targets, and diseases. Moreover, the database contains the pharmacokinetic properties of various chemical components, including their OB and DL. First, Chinese characters such as “Chai Hu” or “Xiang Fu” were entered into the database to determine their respective components and corresponding pharmacokinetic data. Then, the absorption, distribution, metabolism, and excretion (ADME) evaluation system was used to select potential active ingredients. Here, we selected two pharmacokinetic parameters as screening criteria to determine the active ingredients in *Radix Bupleuri* or *Rhizoma Cyperi*, OB ≥30% and DL ≥0.18. Active ingredients that met the criteria were considered candidates for subsequent analysis.

#### Target prediction

We searched the HERB (http://herb.ac.cn/) database for active components of CXP for potential therapeutic target mining to identify targets that bind to CXP active compounds. HERB is a high-throughput experiment- and reference-guided database for TCM. We downloaded GSE60502 from the GEO (http://www.ncbi.nlm.nih.gov/geo/) database to screen for significant DEGs in HCC and identify HCC-related pathological genes. Wang et al. [55] mined and analyzed the gene expression profiles of HCC and adjacent nontumor liver tissues to identify significant DEGs in HCC. The transcriptional results from 18 paired HCC and adjacent nontumorous liver tissues were selected for analysis. Data analysis and graph generation were performed using R version 3.6.3 (https://www.r-project.org). The uniform manifold approximation and projection (UMAP) was analyzed with the ‘umap’ package and visualized with the ‘ggplot2’ package. The DEGs in HCC were screened and obtained using the ‘limma’ package of R language Bioconductor (http://www.bioconductor.org/packages/release/bioc/html/affy.html) with a false discovery rate (FDR) <0.05 and |log2FC| >1. Subsequently, these DEGs were processed using the ‘ggplot2’ package in R language and visualized by constructing a volcano plot. The ‘ComplexHeatmap’ package in R language was used to analyze the top 50 upregulated or downregulated DEGs in HCC. Finally, these genes were compared to obtain the potential overlapping CXP targets and HCC DEGs. These overlapping targets are those that CXP may modulate to treat HCC.

#### Publicly available expression datasets

We downloaded RNA-seq and clinical data from 374 patients with HCC in TCGA (https://portal.gdc.cancer.gov). Fragments per kilobase per million (FPKM) values for TCGA cohort were converted to transcripts per million (TPM) values before further analysis. Subsequently, differences in gene expression between adjacent nontumorous tissues (*n* = 50) and tumor tissues (*n* = 374) were statistically analyzed and visualized using the “ggplot” R package.

### KEGG pathway and GO enrichment analyses

A list of DEGs in HCC or CXP anti-HCC-related targets (species limited to ‘*Homo sapiens*’) was submitted to the OmicShare tool for KEGG pathway and GO enrichment analyses. OmicShare is a free online platform for data analysis (https://www.omicshare.com/tools) that can be used for GO analyses, KEGG analyses, Venn diagrams, heatmaps, network construction, volcano plot analyses, etc.

### Network construction, hub genes, and topology analysis

A list of DEGs in HCC (|log2FC| >1) was input into the STRING database, with the species limited to ‘*Homo sapiens*’ and hidden disconnected nodes (interaction score ≥0.90), to construct a PPI network. Images were exported, and a topological analysis of nodes was performed. A list of DEGs in HCC (|log2FC| >2) or CXP anti-HCC-related targets was submitted to the Metascape webtool (http://metascape.org), with the species limited to ‘*Homo sapiens*’, to construct the PPI network. For each specific gene list, the Metascape platform performed a PPI enrichment analysis using the following databases: STRING, BioGrid, OmniPath, and InWeb_IM. The MCODE algorithm was used to identify densely connected network components if the network contained 3 to 500 proteins. This novel graph-theoretical clustering algorithm, MCODE, may represent densely connected regions in large PPI networks of molecular complexes [56]. We downloaded the raw data for the PPI network obtained from the Metascape analysis and saved the data file in the CYS format. Subsequently, the file was opened using Cytoscape software (version 3.7.2) for visualization, hub gene analysis, and topological analysis. For the hub gene analysis, hub gene networks are available through the CytoHubba plugin in Cytoscape software. Additionally, the parameters of topological features can be calculated using the Cytoscape plugin Network Analyzer, including “Degree”, “Betweenness Centrality”, “Closeness Centrality”, “Clustering Coefficient”, and “Topological Coefficient”. In addition to the PPI network, other networks, such as H-C-T and KEGG pathway-genes, were analyzed and displayed using the OmicShare tool.

### Construction and validation of a prognostic model involving cell cycle-related genes and LASSO Cox regression analysis

Prognostic cell cycle-related genes were identified by performing a univariate Cox analysis of OS of patients included in TCGA-LIHC set using the “survival” package in the R language. The R packages “glmnet” and “survival” were used to perform the LASSO Cox regression analysis with random seeds to build the risk score model that best predicts survival in the training cohort. A risk score formula was then established based on the normalized expression level of each gene and its corresponding regression coefficient: risk score = (0.1159 × CDK4 expression level) + (0.0234 × CHEK1 expression level) + (0.1968 × CCNB1 expression level) + (0.0322 × CDKN2A expression level). The patients were then divided into the low-risk and high-risk groups according to the median risk score.

### Correlation between immune cell infiltration and the expression of cell cycle-related genes

We integrated several computational tools to estimate immune cell infiltration in TCGA RNA-seq cohorts. Based on the centralized algorithms in the online database TIMER2.0 (http://timer.comp-genomics.org/), including the TIMER, EPIC, XCELL, QUANTISEQ, and MCPCOUNTER algorithms, we analyzed the relationship between the expression of cell cycle-related genes (CDK4, CCNB1, CHEK1, and CDKN2A) and immune cell infiltration levels in patients with LIHC. Pearson’s correlation analysis was performed to elucidate the correlations between the expression of cell cycle-related genes expression and immune cell infiltration.

### *In silico* molecular docking study

AutoDock Vina and PyMOL 1.8 software were utilized to perform molecular docking studies of CXP active components and selected proteins. X-ray crystal structures of selected proteins were obtained from the Protein Data Bank (PDB, https://www.rcsb.org/). Then, the PDB file was opened with PyMOL 1.8 to remove water molecules and heteroatoms from the protein crystal structure, add hydrogen atoms, and calculate the charge. Meanwhile, the 3D chemical structures of the active components of CXP were downloaded from PubChem (https://pubchem.ncbi.nlm.nih.gov/) with the format for SDF and converted to a.pdb format by PyMOL 1.8. Next, the proteins and active ingredients were converted into.pdb format files by AutoDockTools (version 1.5.6). Second, the grid box function of AutoDockTools was used to define specific docking pockets in selected proteins that potentially bind to the active ingredient. Finally, the molecular docking analysis was performed using the command prompt, and the docking results were visualized using PyMOL. The binding energy was calculated to assess the theoretical binding affinity of the active ingredient for the selected proteins.

### Statistical analysis

The data analysis and graph generation were performed in R language (version 3.6.3) and with the OmicShare tool. Comparisons between the two groups were performed using the unpaired Student’s *t*-test to analyze the statistical significance of normally distributed variables and the Wilcoxon rank-sum test to estimate the statistical significance of non-normally distributed variables. Kaplan-Meier survival curves for OS or DSS were plotted using the R package “survminer”. ROC curves for 1-, 3-, and 5-year survival were plotted to assess the diagnostic value of risk scores generated using timeROC. *P* < 0.05 was considered statistically significant.

### Data availability

Publicly available datasets were analyzed in this study. This data can be found here: TCMSP, HERB, TCGA, GEO, TIMER2.0, etc.

## Supplementary Materials

Supplementary Tables 1 and 5

Supplementary Tables 2-4 and 6-8
