# Supplementary material for: Exploring the mechanisms underlying the therapeutic effect of the Radix Bupleuri-Rhizoma Cyperi herb pair on hepatocellular carcinoma using multilevel data integration and molecular docking
Source: Aging (Albany NY). 2022 Nov 18;14(22):9103–27. doi: 10.18632/aging.204388 (PMC9740357; doi:10.18632/aging.204388)
Supplement: Supplementary Tables 1 and 5 [file aging-14-204388-s001.docx]

**Supplementary Table 1.** Degree rank of nodes in the PPI network of 1110 DEGs in HCC (Node_degree > 0).

| **Network Node** | **Node_degree** | **Network Node** | **Node_degree** |
| --- | --- | --- | --- |
| CDK1 | 91 | RRS1 | 4 |
| CCNB1 | 72 | RUVBL2 | 4 |
| TOP2A | 62 | SARDH | 4 |
| BUB1 | 61 | SDS | 4 |
| CCNB2 | 59 | SERPINE1 | 4 |
| CDC20 | 58 | SRD5A2 | 4 |
| KIF11 | 56 | TAT | 4 |
| AURKB | 52 | TK1 | 4 |
| BUB1B | 51 | TRAT1 | 4 |
| MAD2L1 | 51 | UPB1 | 4 |
| CDCA8 | 50 | USP1 | 4 |
| ASPM | 49 | A2M | 3 |
| NCAPG | 48 | ACACA | 3 |
| RRM2 | 48 | ACSL1 | 3 |
| NDC80 | 47 | ACSL4 | 3 |
| AURKA | 45 | ACSL5 | 3 |
| DLGAP5 | 45 | ADK | 3 |
| TTK | 44 | AGTR1 | 3 |
| MCM4 | 43 | AKR1B10 | 3 |
| NUSAP1 | 43 | AOC3 | 3 |
| PRC1 | 43 | APOA4 | 3 |
| UBE2C | 43 | APOL1 | 3 |
| CDC45 | 42 | APOM | 3 |
| KIF20A | 42 | ARG1 | 3 |
| MCM3 | 42 | ASL | 3 |
| TPX2 | 41 | ASNS | 3 |
| MCM5 | 39 | ASRGL1 | 3 |
| KIF23 | 38 | BATF | 3 |
| CENPE | 37 | BBOX1 | 3 |
| CEP55 | 37 | BCKDHB | 3 |
| MCM2 | 37 | BDH1 | 3 |
| KIF4A | 36 | BDH2 | 3 |
| MELK | 36 | CD14 | 3 |
| CENPF | 35 | CENPM | 3 |
| CDC6 | 34 | CSPG5 | 3 |
| PBK | 34 | DAK | 3 |
| RFC4 | 34 | EPHA2 | 3 |
| EHHADH | 30 | F13B | 3 |
| PTTG1 | 30 | F9 | 3 |
| RACGAP1 | 29 | FCN2 | 3 |
| CHEK1 | 27 | FCN3 | 3 |
| FOXM1 | 26 | GADD45B | 3 |
| MCM6 | 26 | GADD45G | 3 |
| NCAPH | 26 | GLS2 | 3 |
| RRM1 | 26 | GPT | 3 |
| SMC2 | 26 | GRHPR | 3 |
| ACOX1 | 25 | HBB | 3 |
| CDCA3 | 25 | HK2 | 3 |
| CYP2E1 | 25 | HPR | 3 |
| HJURP | 25 | IRAK3 | 3 |
| KIAA0101 | 24 | LAMA2 | 3 |
| SMC4 | 24 | LUM | 3 |
| TYMS | 24 | MAT1A | 3 |
| CYP3A4 | 23 | MTHFD1 | 3 |
| ECT2 | 22 | MUC13 | 3 |
| MAPK1 | 22 | MUC6 | 3 |
| POLE2 | 22 | NAT2 | 3 |
| CKAP2 | 21 | NOL12 | 3 |
| FYN | 21 | OAT | 3 |
| MCM10 | 21 | PAK1IP1 | 3 |
| AOX1 | 20 | PCK2 | 3 |
| AGXT | 19 | PEMT | 3 |
| CYP1A1 | 19 | PHF21A | 3 |
| CYP2B6 | 19 | PKN1 | 3 |
| F2 | 19 | PLA2G16 | 3 |
| HMGCL | 19 | PTPN22 | 3 |
| PRIM1 | 19 | RCL1 | 3 |
| CDKN3 | 18 | SETDB1 | 3 |
| CKS2 | 18 | STMN1 | 3 |
| FEN1 | 18 | UBE2M | 3 |
| GINS2 | 18 | ACD | 2 |
| CYP1A2 | 17 | AFP | 2 |
| CYP2C9 | 17 | ALDH1L1 | 2 |
| CYP4A11 | 17 | ANGPT2 | 2 |
| LCK | 17 | BCAT1 | 2 |
| APOA1 | 16 | CBX1 | 2 |
| EPHX2 | 16 | CCL19 | 2 |
| ESR1 | 16 | CCL20 | 2 |
| GINS1 | 16 | CCNF | 2 |
| MYC | 16 | CCT6A | 2 |
| ACOX2 | 15 | CD163 | 2 |
| CAT | 15 | CD1D | 2 |
| CYP26A1 | 15 | CDA | 2 |
| CYP2A6 | 15 | CDO1 | 2 |
| DAO | 15 | CES2 | 2 |
| GMNN | 15 | CHST4 | 2 |
| H2AFZ | 15 | COL5A3 | 2 |
| KIFC1 | 15 | COLEC11 | 2 |
| OIP5 | 15 | CPT2 | 2 |
| PRIM2 | 15 | CSAD | 2 |
| TACC3 | 15 | CYP2C18 | 2 |
| TIPIN | 15 | DBH | 2 |
| DTL | 14 | DCLRE1B | 2 |
| EZH2 | 14 | DCTPP1 | 2 |
| FOS | 14 | DKK1 | 2 |
| CDK4 | 13 | DUSP4 | 2 |
| CYP2C8 | 13 | DUSP9 | 2 |
| GINS3 | 13 | EDNRB | 2 |
| HAO1 | 13 | ERCC2 | 2 |
| HAO2 | 13 | ERCC6L | 2 |
| NUP62 | 13 | FCGR2B | 2 |
| ORC6 | 13 | FGF16 | 2 |
| SLC27A2 | 13 | FMO3 | 2 |
| AKR1C3 | 12 | FMO4 | 2 |
| ALDH2 | 12 | FMO5 | 2 |
| CCNE1 | 12 | FOSL1 | 2 |
| CD4 | 12 | FTCD | 2 |
| CYP2C19 | 12 | GCNT3 | 2 |
| CYP2J2 | 12 | GHR | 2 |
| GINS4 | 12 | GLRX | 2 |
| MPV17 | 12 | GSTZ1 | 2 |
| NRAS | 12 | HAAO | 2 |
| NUP155 | 12 | HAMP | 2 |
| NUP93 | 12 | HELLS | 2 |
| PECR | 12 | HGD | 2 |
| CXCL12 | 11 | HMOX1 | 2 |
| FOXO1 | 11 | HPD | 2 |
| IGF1 | 11 | HPX | 2 |
| KIF14 | 11 | HRG | 2 |
| PKM | 11 | HS2ST1 | 2 |
| PTGS2 | 11 | IL1RL1 | 2 |
| SNRPD1 | 11 | IL33 | 2 |
| ACADL | 10 | IL7R | 2 |
| ACAT1 | 10 | ITIH4 | 2 |
| ALDH1A3 | 10 | KMO | 2 |
| ALDH6A1 | 10 | LIPC | 2 |
| BARD1 | 10 | LOX | 2 |
| CCL4 | 10 | MGA | 2 |
| CDK5 | 10 | MMP12 | 2 |
| CYP3A7 | 10 | MTTP | 2 |
| ENPP1 | 10 | MYCN | 2 |
| H2AFX | 10 | NCF1 | 2 |
| HIST1H2BO | 10 | NGFR | 2 |
| HSD17B6 | 10 | NR1I3 | 2 |
| MSH2 | 10 | NTF3 | 2 |
| NUP43 | 10 | PCOLCE | 2 |
| PLG | 10 | PINK1 | 2 |
| RAN | 10 | PSPH | 2 |
| SP1 | 10 | PTDSS2 | 2 |
| ZWINT | 10 | PTPN13 | 2 |
| AAAS | 9 | PYGB | 2 |
| ACAA2 | 9 | RBP1 | 2 |
| ADH1B | 9 | RELN | 2 |
| F12 | 9 | SERPINF2 | 2 |
| H2BFS | 9 | SERPINH1 | 2 |
| HIST1H2BH | 9 | SF3A2 | 2 |
| KIF18A | 9 | SNRPC | 2 |
| KPNA2 | 9 | SOCS2 | 2 |
| PAK1 | 9 | SULT2A1 | 2 |
| SERPINC1 | 9 | TMPO | 2 |
| THBS1 | 9 | ABCA6 | 1 |
| ACAA1 | 8 | ABCB11 | 1 |
| ARRB2 | 8 | ADAM15 | 1 |
| ASS1 | 8 | AGPAT4 | 1 |
| CD247 | 8 | AKAP12 | 1 |
| CD8A | 8 | ALAD | 1 |
| CYP4F2 | 8 | ANK3 | 1 |
| CYP4F3 | 8 | AP1M2 | 1 |
| EHMT2 | 8 | APOF | 1 |
| GPC3 | 8 | ASPH | 1 |
| LMNB1 | 8 | ATAD2 | 1 |
| PLK4 | 8 | ATP1A1 | 1 |
| PTPRC | 8 | CBLC | 1 |
| SDC3 | 8 | CCL16 | 1 |
| SERPING1 | 8 | CCL21 | 1 |
| UBE2S | 8 | CD244 | 1 |
| ZWILCH | 8 | CDKN2C | 1 |
| ACADSB | 7 | CDYL | 1 |
| AKR1D1 | 7 | CHI3L1 | 1 |
| ALDOA | 7 | CIITA | 1 |
| CAD | 7 | CKAP4 | 1 |
| CCL2 | 7 | CLEC4M | 1 |
| CDC25C | 7 | COPS7B | 1 |
| CHAF1A | 7 | CRLF2 | 1 |
| CHD3 | 7 | CYFIP2 | 1 |
| E2F5 | 7 | CYP27A1 | 1 |
| F11 | 7 | DCXR | 1 |
| FPR1 | 7 | DHODH | 1 |
| HSD17B2 | 7 | DHTKD1 | 1 |
| IRAK1 | 7 | DIO1 | 1 |
| MBL2 | 7 | DNAJC6 | 1 |
| PES1 | 7 | DNMT3L | 1 |
| PROC | 7 | DONSON | 1 |
| AACS | 6 | DPT | 1 |
| ABAT | 6 | DPYS | 1 |
| ADRB2 | 6 | ENPEP | 1 |
| AR | 6 | EXOC3 | 1 |
| CBX4 | 6 | EXOC5 | 1 |
| CBX5 | 6 | FBLN5 | 1 |
| CBX8 | 6 | FGF13 | 1 |
| CETP | 6 | FGL2 | 1 |
| CFP | 6 | FKBP10 | 1 |
| COL4A2 | 6 | FOLH1 | 1 |
| COMT | 6 | FXYD1 | 1 |
| DCN | 6 | GCH1 | 1 |
| DSCC1 | 6 | GDNF | 1 |
| E2F6 | 6 | GGT5 | 1 |
| E2F8 | 6 | GNAL | 1 |
| ECHS1 | 6 | GPD1 | 1 |
| FANCG | 6 | GREB1 | 1 |
| FGFR2 | 6 | GRIN2D | 1 |
| HMGCS2 | 6 | GYS2 | 1 |
| HP | 6 | HIST1H2AM | 1 |
| IGFALS | 6 | HIST1H2BE | 1 |
| IGFBP3 | 6 | HMGA1 | 1 |
| KIF18B | 6 | HSPB1 | 1 |
| LCAT | 6 | HTRA2 | 1 |
| LCP2 | 6 | ID1 | 1 |
| LEF1 | 6 | ID3 | 1 |
| LPA | 6 | IFIT1 | 1 |
| MASP1 | 6 | IGFBP2 | 1 |
| MASP2 | 6 | IGFBP4 | 1 |
| MMP1 | 6 | IGFBP6 | 1 |
| MNAT1 | 6 | IHH | 1 |
| PHC1 | 6 | IL13RA2 | 1 |
| PLCB1 | 6 | IL18R1 | 1 |
| RRP9 | 6 | IL18RAP | 1 |
| SHCBP1 | 6 | KAZN | 1 |
| SPC25 | 6 | KCNMB3 | 1 |
| SPDL1 | 6 | KDM8 | 1 |
| SULT1A1 | 6 | KLHL12 | 1 |
| ADH1A | 5 | KLHL2 | 1 |
| ADH1C | 5 | KRI1 | 1 |
| ADH6 | 5 | LHX2 | 1 |
| ALAS1 | 5 | LILRB2 | 1 |
| ALDH1B1 | 5 | LMNB2 | 1 |
| ALPL | 5 | LYVE1 | 1 |
| ASPA | 5 | MEFV | 1 |
| BCL9 | 5 | MGAM | 1 |
| C6 | 5 | MLST8 | 1 |
| C7 | 5 | MMP11 | 1 |
| CENPU | 5 | MRC1 | 1 |
| COL4A1 | 5 | MT1G | 1 |
| COL4A5 | 5 | MT1H | 1 |
| CPS1 | 5 | NEB | 1 |
| CTH | 5 | NFE2 | 1 |
| CXCR2 | 5 | NKG7 | 1 |
| DSN1 | 5 | NMRK1 | 1 |
| EGR1 | 5 | NPHS2 | 1 |
| ENO3 | 5 | NQO1 | 1 |
| F8 | 5 | NR1I2 | 1 |
| FANCF | 5 | NSUN6 | 1 |
| GCDH | 5 | OGDHL | 1 |
| GCKR | 5 | PAFAH1B3 | 1 |
| GIT2 | 5 | PDE2A | 1 |
| GLUD1 | 5 | PDE4B | 1 |
| GNA14 | 5 | PDGFRA | 1 |
| HSD11B1 | 5 | PFDN4 | 1 |
| IL1RAP | 5 | PGAM2 | 1 |
| IL1RN | 5 | PIK3C2G | 1 |
| KLKB1 | 5 | PPAP2B | 1 |
| LAMA3 | 5 | PPCDC | 1 |
| NASP | 5 | PPIH | 1 |
| OTC | 5 | PPL | 1 |
| PON1 | 5 | PRKG1 | 1 |
| SRD5A1 | 5 | PROZ | 1 |
| SRD5A3 | 5 | PTGIS | 1 |
| TEK | 5 | PTH1R | 1 |
| TKT | 5 | PTPRB | 1 |
| WDR46 | 5 | RFX5 | 1 |
| XYLT2 | 5 | ROBO1 | 1 |
| ACLY | 4 | RRAGD | 1 |
| ADAMTS1 | 4 | S100A8 | 1 |
| ADAMTSL2 | 4 | SC5D | 1 |
| ADAMTSL3 | 4 | SCN2A | 1 |
| ALDOB | 4 | SERPINA10 | 1 |
| BGN | 4 | SHBG | 1 |
| BHMT | 4 | SLC10A1 | 1 |
| BHMT2 | 4 | SLC22A1 | 1 |
| C1QB | 4 | SLC27A5 | 1 |
| C1R | 4 | SLC47A1 | 1 |
| C1S | 4 | SMARCAL1 | 1 |
| C8A | 4 | SMYD3 | 1 |
| C8B | 4 | SOX4 | 1 |
| C9 | 4 | SPN | 1 |
| CCR1 | 4 | SPP1 | 1 |
| CDKN2A | 4 | SPTBN2 | 1 |
| CHAF1B | 4 | SQLE | 1 |
| CXCL2 | 4 | ST3GAL6 | 1 |
| DTYMK | 4 | STAB2 | 1 |
| EPHA3 | 4 | STIP1 | 1 |
| FBP1 | 4 | SYNE1 | 1 |
| FOSB | 4 | TBCC | 1 |
| G6PD | 4 | TBCD | 1 |
| GNMT | 4 | TBXAS1 | 1 |
| GSTM1 | 4 | TDO2 | 1 |
| GZMH | 4 | TFR2 | 1 |
| KNG1 | 4 | TPM2 | 1 |
| KNTC1 | 4 | TRDN | 1 |
| MYBL2 | 4 | TREH | 1 |
| PCK1 | 4 | TTR | 1 |
| PIN1 | 4 | USP39 | 1 |
| POLR2K | 4 | VRK1 | 1 |
| PRMT5 | 4 | VSIG4 | 1 |
| RAD51AP1 | 4 | WDR18 | 1 |
| RDH16 | 4 | ZMAT5 | 1 |

**Supplementary Table S5:** Therapeutic targets of *Radix Bupleuri* (196), *Rhizoma Cyperi* (159), and 1110 DEGs of HCC.

| **HCC DEGs** | ***Radix Bupleuri*** | ***Rhizoma Cyperi*** |
| --- | --- | --- |
| UPK3A | ACHE | AR |
| CCL20 | AHR | CALM3 |
| MAGEA6 | AKT1 | CDK2 |
| TMEM156 | ALOX5 | CYP1A1 |
| NAT8B | AR | CYP1A2 |
| ECHDC3 | BAX | ESR1 |
| FOSL1 | BCL2 | GSK3B |
| NQO1 | CALM3 | ABCC1 |
| IRAK3 | CASP3 | NOS2 |
| POPDC3 | CHRM1 | PRSS1 |
| TRAT1 | CYP1A1 | PTGS1 |
| C11orf80 | CYP1A2 | PTGS2 |
| DNALI1 | CYP1B1 | NCOA1 |
| GSTM1 | CYP3A4 | PIM1 |
| TMC5 | DIO1 | PPARG |
| AFP | ESR1 | CHEK1 |
| C17orf75 | ESR2 | NCOA2 |
| CHI3L1 | GSTM1 | DPP4 |
| PGAM2 | GSTM2 | CCNA2 |
| SLC47A1 | GSTP1 | MAPK14 |
| DIO1 | HAS2 | CA2 |
| CCL25 | HMOX1 | DPEP1 |
| TBXAS1 | ICAM1 | ESR2 |
| PODXL2 | IKBKB | OLR1 |
| FAP | INSR | RELA |
| ATP13A2 | JUN | XDH |
| VNN2 | MMP1 | NCF1 |
| PTPN22 | NOS2 | PPARD |
| HPGD | PGR | F7 |
| MYC | POLD1 | MAOB |
| ID3 | PPARG | GABRA1 |
| CPT2 | PPP3CA | PYGM |
| PDK4 | MAPK8 | AKR1B1 |
| LEF1 | PRSS1 | GRIA2 |
| ARRB2 | PSMD3 | ACHE |
| NXF3 | PTGS1 | KCNH2 |
| REG3A | PTGS2 | KDR |
| FGFR2 | RELA | RXRA |
| SMPX | SELE | SCN5A |
| PAK1IP1 | SLC2A4 | PDE3A |
| LUM | SLC6A2 | BAX |
| KNG1 | SLPI | BCL2 |
| BANK1 | STAT1 | CASP3 |
| PTGFR | TNF | CASP8 |
| RRS1 | VCAM1 | CASP9 |
| AKR1B10 | XDH | JUN |
| FBXO17 | AKR1C3 | MAP2 |
| IGFBP2 | NR1I2 | PON1 |
| MFAP4 | NR1I3 | PRKCA |
| IL1RAP | AHSA1 | TGFB1 |
| KIAA1731 | F7 | CHRM4 |
| SOAT2 | GABRA1 | DRD1 |
| FKBP10 | PIM1 | NR3C1 |
| PDK1 | MAPK14 | CHRM1 |
| ARHGAP4 | CDK1 | CHRNA2 |
| ABCC2 | NCOA2 | CHRNA7 |
| PDE9A | DPEP1 | CHRM3 |
| CNNM4 | CDK2 | PGR |
| ANGPT2 | ADRA1B | ADRB2 |
| PDE4B | GSK3B | SLC6A4 |
| ADAMTSL2 | CHRM2 | NR3C2 |
| LINC00328 | CCNA2 | OPRM1 |
| COMP | CA2 | ADRA1B |
| ZNF157 | CHEK1 | CHRM2 |
| FER | DPP4 | ADRA1D |
| DKK1 | KCNH2 | APOE |
| ZFP37 | NR3C1 | DHCR24 |
| IHH | NR3C2 | ICAM1 |
| MFSD6 | ADRB2 | SREBF1 |
| COL14A1 | SCN5A | SREBF2 |
| SERPINE1 | NCOA1 | ABCB11 |
| IGFBP4 | ACACA | ADRB1 |
| E2F5 | ACP3 | DRD2 |
| CA9 | PARP1 | DRD3 |
| IGLL3P | AKR1B1 | HTR3A |
| SLC10A3 | BIRC5 | OPRD1 |
| PAK1 | CCND1 | RXRB |
| MYCN | BCL2L1 | SLC6A2 |
| CFHR5 | BMP2 | SLC6A3 |
| CWH43 | CASP8 | ADRA2C |
| LY6G6E | CASP9 | CHRM5 |
| NUP62CL | CAT | AHR |
| CA2 | CAV1 | AKT1 |
| NPHS2 | RUNX2 | ALOX5 |
| MYL10 | RUNX1T1 | CYP1B1 |
| IGF2BP2 | CCNB1 | CYP3A4 |
| COX7A1 | CD40LG | DIO1 |
| FMO5 | CDKN1A | GSTM1 |
| PEG10 | CDKN2A | GSTM2 |
| RBP1 | CHUK | GSTP1 |
| GSTA1 | COL1A1 | HAS2 |
| AOAH | COL3A1 | HMOX1 |
| NECAB2 | CLDN4 | IKBKB |
| SERPINC1 | CRP | INSR |
| MMP12 | CTSD | MMP1 |
| GAS1 | NQO1 | POLD1 |
| GUSBP11 | E2F1 | PPP3CA |
| PRG2 | E2F2 | MAPK8 |
| ALDH1A3 | EGF | PSMD3 |
| KIF18A | EGFR | SELE |
| ID4 | ELK1 | SLC2A4 |
| BHMT2 | ERBB2 | SLPI |
| GIT2 | ERBB3 | STAT1 |
| F13B | F3 | TNF |
| ANGPTL4 | FOS | VCAM1 |
| LAMA3 | GJA1 | AKR1C3 |
| CYFIP2 | CXCL2 | NR1I2 |
| COL4A5 | HIF1A | NR1I3 |
| ASPN | HK2 | AHSA1 |
| TACSTD2 | HSF1 | CDK1 |
| ETV4 | HSPA5 | ABCA1 |
| CES1P1 | HSPB1 | HMGCR |
| TRDN | IFNG | SLCO1B1 |
| PPCDC | IGF2 | ABCG5 |
| APOM | IGFBP3 | ABCG8 |
| ALPK3 | IL1A | LTA4H |
| RAD54B | IL1B | MAOA |
| SSX1 | IL2 | CTRB1 |
| RAB38 | IL6 | PLAU |
| HIST1H2BH | CXCL8 | ADRA2A |
| APCS | IL10 | ADH1C |
| PLG | CXCL10 | HTR2A |
| CCDC144A | IRF1 | ADRA1A |
| COL5A3 | EIF6 | GABRA3 |
| SSSCA1 | MAOB | ADCY2 |
| ZNF696 | MMP2 | XIAP |
| TNFSF14 | MMP3 | BIRC5 |
| ENPEP | MMP9 | APP |
| IL1RN | MPO | CCND1 |
| BCAT1 | MYC | BCL2L1 |
| SCN2A | NFE2L2 | CASP7 |
| GREM2 | NFKBIA | CCNB1 |
| SV2B | NKX3-1 | CD40LG |
| TFPI2 | NOS3 | CDK4 |
| ASPH | ODC1 | CDKN1A |
| GREB1 | SERPINE1 | EGFR |
| GRIN2D | PCOLCE | ERBB2 |
| DOCK2 | PLAT | IFNG |
| DUOX2 | PLAU | IL2 |
| KCNE1L | PON1 | IL4 |
| NFE2 | POR | IL6 |
| CFD | PPARA | IL10 |
| CGREF1 | PPARD | MCL1 |
| BGN | PRKCA | MDM2 |
| SPAG4 | PRKCB | MET |
| HIST1H2AM | MAPK1 | MMP2 |
| NCF1 | MAPK3 | MMP9 |
| LAMA2 | PTEN | NFKBIA |
| RUNDC3B | PTGER3 | PCNA |
| GSTA3 | RAF1 | MAPK1 |
| ADAMTS1 | RASA1 | MAPK3 |
| SRD5A3 | RB1 | RB1 |
| SMARCAL1 | RXRA | TOP1 |
| MUC6 | CCL2 | TOP2A |
| MMP1 | CXCL11 | TP53 |
| CFHR2 | SOD1 | TYR |
| C1QB | SOD3 | VEGFA |
| ERCC6L | SPP1 | PTGES |
| FHL5 | SULT1E1 | NUF2 |
| IGLJ3 | TGFB1 |  |
| CXCL2 | THBD |  |
| KCNMB3 | TOP1 |  |
| AKAP12 | TOP2A |  |
| OR1E1 | TP53 |  |
| ZCWPW1 | VEGFA |  |
| SDS | DCAF5 |  |
| GPR20 | MGAM |  |
| SLC1A1 | ABCG2 |  |
| MCOLN3 | NPEPPS |  |
| SLC17A3 | ACAA2 |  |
| RIBC2 | RASSF1 |  |
| GHSR | CHEK2 |  |
| FOXRED2 | DUOX2 |  |
| MGAM | HERC5 |  |
| HIST1H2BO | NCF1 |  |
| ANO1 | ABCA1 |  |
| APOA4 | HMGCR |  |
| MEG3 | SLCO1B1 |  |
| CYP4F3 | ABCG5 |  |
| MNX1 | ABCG8 |  |
| PTGIS | LTA4H |  |
| GMDS | MAOA |  |
| FOXE1 | CTRB1 |  |
| PHC1 | SLC6A3 |  |
| ZNF532 | ADRA1D |  |
| SLC4A2 | CHRNA7 |  |
| AP1M2 | ADRB1 |  |
| PYGB | ADRA2A |  |
| ASIC1 | CHRM3 |  |
| IL1RL1 | OLR1 |  |
| WDR18 | PYGM |  |
| PSPH | GRIA2 |  |
| RNF41 | ADH1C |  |
| AGXT | HTR2A |  |
| G0S2 | ADRA1A |  |
| SLC2A2 | GABRA3 |  |
| DUSP4 |  |  |
| TPD52L2 |  |  |
| SLC17A2 |  |  |
| MBNL2 |  |  |
| KLHL12 |  |  |
| TFPI |  |  |
| SVEP1 |  |  |
| SLC37A1 |  |  |
| DNAJB6 |  |  |
| CD320 |  |  |
| DPYS |  |  |
| IGLV1-44 |  |  |
| LGALSL |  |  |
| MXRA5 |  |  |
| PIN1 |  |  |
| PPL |  |  |
| TRIM22 |  |  |
| TSKU |  |  |
| LIPC |  |  |
| TMOD3 |  |  |
| APOL2 |  |  |
| ALDH1L1 |  |  |
| AGPAT4 |  |  |
| GIMAP6 |  |  |
| HMGCS2 |  |  |
| NOL8 |  |  |
| PTPRC |  |  |
| SLC6A6 |  |  |
| PCTP |  |  |
| ZBTB6 |  |  |
| GPR6 |  |  |
| FBLN5 |  |  |
| IL7R |  |  |
| FNDC4 |  |  |
| HRSP12 |  |  |
| ZNF385D |  |  |
| SLC27A2 |  |  |
| RGP1 |  |  |
| BTBD3 |  |  |
| PTPN13 |  |  |
| GLUD1 |  |  |
| TRIM16 |  |  |
| ZNF580 |  |  |
| AASS |  |  |
| HSD17B6 |  |  |
| CIITA |  |  |
| AIF1 |  |  |
| ALG12 |  |  |
| TKT |  |  |
| SLC38A4 |  |  |
| ZFHX3 |  |  |
| ZMAT5 |  |  |
| SNX17 |  |  |
| EPHA3 |  |  |
| PRMT5 |  |  |
| TWSG1 |  |  |
| ALDOB |  |  |
| HK2 |  |  |
| ADIRF |  |  |
| SCGN |  |  |
| LCK |  |  |
| MPP6 |  |  |
| CRISPLD2 |  |  |
| GMFG |  |  |
| BDH1 |  |  |
| IGFBP6 |  |  |
| NVL |  |  |
| SPN |  |  |
| GYPC |  |  |
| GBP1 |  |  |
| NEB |  |  |
| CXorf57 |  |  |
| TRBC1 |  |  |
| CDO1 |  |  |
| MME |  |  |
| HTR1A |  |  |
| TREH |  |  |
| GFI1B |  |  |
| LHX2 |  |  |
| IGK |  |  |
| ZNF165 |  |  |
| SLC22A7 |  |  |
| FOLR2 |  |  |
| MEP1A |  |  |
| CA5A |  |  |
| FPR1 |  |  |
| SAMSN1 |  |  |
| ITIH1 |  |  |
| GCNT3 |  |  |
| IGHD |  |  |
| FAIM3 |  |  |
| ACOX2 |  |  |
| STK39 |  |  |
| LGSN |  |  |
| XYLT2 |  |  |
| S100A8 |  |  |
| TTLL1 |  |  |
| RGN |  |  |
| UGT2B15 |  |  |
| PHF21A |  |  |
| LINC00652 | |  |
| ZFP36 |  |  |
| HRG |  |  |
| NF2 |  |  |
| C8B |  |  |
| ZNF184 |  |  |
| UGT2B28 |  |  |
| LINC00094 | |  |
| RAB23 |  |  |
| HIC2 |  |  |
| CCDC94 |  |  |
| RGS5 |  |  |
| SLC6A8 |  |  |
| ERCC2 |  |  |
| DACH1 |  |  |
| CCL16 |  |  |
| ARG1 |  |  |
| TPM2 |  |  |
| FGL2 |  |  |
| MFSD10 |  |  |
| DNAJC10 |  |  |
| LPAL2 |  |  |
| MOGAT2 |  |  |
| IL18RAP |  |  |
| KBTBD11 |  |  |
| GPATCH1 | |  |
| CD24 |  |  |
| CCL23 |  |  |
| IFIT1 |  |  |
| CSPG5 |  |  |
| MMP11 |  |  |
| APBA2 |  |  |
| CPS1 |  |  |
| ABCB4 |  |  |
| BBOX1 |  |  |
| TIPIN |  |  |
| RASAL2 |  |  |
| CES2 |  |  |
| ASNS |  |  |
| ANKEF1 |  |  |
| SENP7 |  |  |
| CYP2C18 |  |  |
| HYAL1 |  |  |
| PECR |  |  |
| MLLT11 |  |  |
| UPB1 |  |  |
| OAT |  |  |
| LCP2 |  |  |
| ACACA |  |  |
| RPL39L |  |  |
| NPR3 |  |  |
| ABCB11 |  |  |
| AQP9 |  |  |
| IL33 |  |  |
| ANGPTL3 |  |  |
| CD244 |  |  |
| HGD |  |  |
| IGKC |  |  |
| NKG7 |  |  |
| PTTG3P |  |  |
| IGHG1 |  |  |
| PFKFB1 |  |  |
| P2RY13 |  |  |
| HIST1H2BE | |  |
| HSD17B2 |  |  |
| C1S |  |  |
| BATF |  |  |
| FKBPL |  |  |
| SLC1A2 |  |  |
| CYP4F2 |  |  |
| CBLC |  |  |
| CEP131 |  |  |
| AR |  |  |
| CD55 |  |  |
| LEPREL1 |  |  |
| SPP1 |  |  |
| FTCD |  |  |
| GAL3ST1 |  |  |
| PTPRB |  |  |
| CST7 |  |  |
| ADRB2 |  |  |
| ROBO1 |  |  |
| DCTPP1 |  |  |
| RP1-8B22.1 | |  |
| C4BPA |  |  |
| TK1 |  |  |
| HP |  |  |
| DSCC1 |  |  |
| CD69 |  |  |
| IGLC1 |  |  |
| AOX1 |  |  |
| WDR46 |  |  |
| ASAP3 |  |  |
| FHL1 |  |  |
| STEAP4 |  |  |
| CYP4F12 |  |  |
| ACSL1 |  |  |
| KRT16 |  |  |
| TTR |  |  |
| TAGLN2 |  |  |
| FGF13 |  |  |
| MGA |  |  |
| G6PD |  |  |
| PDGFRA |  |  |
| EXOC5 |  |  |
| NDRG1 |  |  |
| ANKZF1 |  |  |
| ALPL |  |  |
| STMN1 |  |  |
| ZFPM2 |  |  |
| NCLN |  |  |
| COL4A2 |  |  |
| XK |  |  |
| ANXA10 |  |  |
| CHAF1B |  |  |
| DAO |  |  |
| SOX4 |  |  |
| CAD |  |  |
| PIK3C2G |  |  |
| EMR2 |  |  |
| HAND2-AS1 | |  |
| ACSL5 |  |  |
| CCL21 |  |  |
| WFDC1 |  |  |
| PRKG1 |  |  |
| EVI2B |  |  |
| GGT5 |  |  |
| PCK1 |  |  |
| 4-Sep |  |  |
| GLRX |  |  |
| PCDH9 |  |  |
| FAM149A | |  |
| F2 |  |  |
| OTC |  |  |
| PRAF2 |  |  |
| PAIP2B |  |  |
| LAG3 |  |  |
| THBS1 |  |  |
| GINS3 |  |  |
| EXOG |  |  |
| CXCR2 |  |  |
| MPV17 |  |  |
| SLC39A2 |  |  |
| CRLF2 |  |  |
| H2BFS |  |  |
| FANCF |  |  |
| CHD3 |  |  |
| PRODH2 |  |  |
| MYO1F |  |  |
| TENM1 |  |  |
| S100P |  |  |
| PCSK1N |  |  |
| CLCA3P |  |  |
| SLCO1B1 |  |  |
| FKBP11 |  |  |
| LMNB2 |  |  |
| C9 |  |  |
| SLC28A1 |  |  |
| LCE2B |  |  |
| CWF19L1 |  |  |
| TFR2 |  |  |
| FETUB |  |  |
| PTDSS2 |  |  |
| SETDB1 |  |  |
| E2F6 |  |  |
| PON1 |  |  |
| CYP2C19 |  |  |
| ENPP1 |  |  |
| DPP3 |  |  |
| PDGFRL |  |  |
| MEFV |  |  |
| NPC1L1 |  |  |
| LILRB2 |  |  |
| POU3F2 |  |  |
| EXOC3 |  |  |
| SMAD6 |  |  |
| TCFL5 |  |  |
| CCNF |  |  |
| KNOP1 |  |  |
| DCLRE1B |  |  |
| KCNAB1 |  |  |
| HAO2 |  |  |
| NT5DC2 |  |  |
| KCNJ8 |  |  |
| CCL4 |  |  |
| BARD1 |  |  |
| PIK3C2B |  |  |
| SULT2A1 |  |  |
| SERPING1 | |  |
| DNMT3L |  |  |
| HPR |  |  |
| ADCK2 |  |  |
| TRMT2A |  |  |
| TP53I3 |  |  |
| MPDZ |  |  |
| TOP3B |  |  |
| TTC39A |  |  |
| ADAM15 |  |  |
| TMEM100 | |  |
| AKR1C3 |  |  |
| A2M |  |  |
| GTPBP2 |  |  |
| ANK3 |  |  |
| ZNF3 |  |  |
| ASGR1 |  |  |
| CFHR4 |  |  |
| CYR61 |  |  |
| SP1 |  |  |
| CD247 |  |  |
| SDC3 |  |  |
| KCND3 |  |  |
| ITIH4 |  |  |
| GPR65 |  |  |
| SMYD5 |  |  |
| DHODH |  |  |
| IL18R1 |  |  |
| TMEM104 | |  |
| HMOX1 |  |  |
| ALDH1B1 |  |  |
| TRAV12-2 | |  |
| LEPREL4 |  |  |
| GDNF |  |  |
| RRP9 |  |  |
| MCM5 |  |  |
| PES1 |  |  |
| TBCC |  |  |
| NTF3 |  |  |
| TEK |  |  |
| NR1I2 |  |  |
| MASP1 |  |  |
| CBX4 |  |  |
| GADD45B | |  |
| SLC46A3 |  |  |
| PRSS53 |  |  |
| NASP |  |  |
| FMO3 |  |  |
| NUP93 |  |  |
| ABLIM3 |  |  |
| SHBG |  |  |
| GLS2 |  |  |
| SATB1 |  |  |
| RAD51AP1 | |  |
| F12 |  |  |
| SEC14L2 |  |  |
| ZC3H13 |  |  |
| CDC25C |  |  |
| MTTP |  |  |
| CNGA1 |  |  |
| BLMH |  |  |
| TCF21 |  |  |
| MYRIP |  |  |
| CYP2B7P |  |  |
| NDRG3 |  |  |
| FGF16 |  |  |
| SLC23A2 |  |  |
| PLIN1 |  |  |
| KDM8 |  |  |
| GCH1 |  |  |
| CBX5 |  |  |
| ANG |  |  |
| YKT6 |  |  |
| SRD5A1 |  |  |
| POGK |  |  |
| ENO3 |  |  |
| SPATA6L |  |  |
| AAAS |  |  |
| MUC13 |  |  |
| SPTBN2 |  |  |
| APOL1 |  |  |
| NOL12 |  |  |
| ASL |  |  |
| CCL2 |  |  |
| CYP27A1 |  |  |
| IGF1 |  |  |
| LST1 |  |  |
| NUP155 |  |  |
| NUP43 |  |  |
| AKIP1 |  |  |
| SULT1A1 |  |  |
| PLK4 |  |  |
| CASP4 |  |  |
| PRG4 |  |  |
| EGR1 |  |  |
| SUSD4 |  |  |
| KIFC1 |  |  |
| PKM |  |  |
| CYP2J2 |  |  |
| SARDH |  |  |
| FOXO1 |  |  |
| EDNRB |  |  |
| HCG9 |  |  |
| FMO4 |  |  |
| CDYL |  |  |
| RELN |  |  |
| ACSM3 |  |  |
| ALLC |  |  |
| ACD |  |  |
| EHMT2 |  |  |
| HMGN1 |  |  |
| GFOD2 |  |  |
| APBA1 |  |  |
| MBL2 |  |  |
| VNN1 |  |  |
| SQLE |  |  |
| SLC39A6 |  |  |
| OLFML3 |  |  |
| LDB2 |  |  |
| IGSF3 |  |  |
| SERPINA10 | |  |
| C8orf4 |  |  |
| CIZ1 |  |  |
| COBLL1 |  |  |
| AKR7A3 |  |  |
| RFX5 |  |  |
| REXO4 |  |  |
| LECT2 |  |  |
| VRK1 |  |  |
| SERPINF2 |  |  |
| ASPA |  |  |
| HAO1 |  |  |
| C1orf106 |  |  |
| ALDH6A1 |  |  |
| CPN2 |  |  |
| IL10RA |  |  |
| PTGS2 |  |  |
| IGJ |  |  |
| TBCD |  |  |
| RHBDD3 |  |  |
| COPS7B |  |  |
| CHAF1A |  |  |
| DNAJC12 |  |  |
| F9 |  |  |
| TAT |  |  |
| PROZ |  |  |
| IL13RA2 |  |  |
| DHTKD1 |  |  |
| RNF125 |  |  |
| ADAMTSL3 | |  |
| FAM13A |  |  |
| MCM10 |  |  |
| CTSO |  |  |
| LYZL6 |  |  |
| CD163 |  |  |
| NABP2 |  |  |
| ADH1B |  |  |
| KRI1 |  |  |
| AURKB |  |  |
| HCLS1 |  |  |
| GNAL |  |  |
| CYP4A11 |  |  |
| DIRAS3 |  |  |
| NDUFA7 |  |  |
| TMCO3 |  |  |
| BCL9 |  |  |
| MPHOSPH9 | |  |
| CKS2 |  |  |
| RRAGD |  |  |
| PLA2G16 |  |  |
| GOLPH3L |  |  |
| MMRN1 |  |  |
| LOC81691 | |  |
| DLEU2 |  |  |
| RUVBL2 |  |  |
| CCL19 |  |  |
| DUSP9 |  |  |
| HPD |  |  |
| C1RL |  |  |
| USP1 |  |  |
| CCNE1 |  |  |
| CENPE |  |  |
| HLF |  |  |
| ZFYVE26 |  |  |
| MLST8 |  |  |
| ID1 |  |  |
| ECHDC2 |  |  |
| TTC38 |  |  |
| GPR126 |  |  |
| GZMK |  |  |
| PCOLCE |  |  |
| OGDHL |  |  |
| ETNPPL |  |  |
| ACADSB |  |  |
| AGBL5 |  |  |
| NCAPH |  |  |
| BCHE |  |  |
| GPT |  |  |
| CYP3A43 |  |  |
| CDCA8 |  |  |
| LAGE3 |  |  |
| TDO2 |  |  |
| C8A |  |  |
| PLCE1 |  |  |
| COMT |  |  |
| HPX |  |  |
| ADK |  |  |
| HGFAC |  |  |
| ZCCHC24 |  |  |
| BCKDHB |  |  |
| CYP3A7-CYP3AP1 | |  |
| PRIM1 |  |  |
| GRHPR |  |  |
| TMEM251 | |  |
| AZGP1 |  |  |
| PIGC |  |  |
| DLC1 |  |  |
| PRR7 |  |  |
| LOC157562 | |  |
| MNAT1 |  |  |
| CENPU |  |  |
| FAM49B |  |  |
| CKAP2 |  |  |
| APOA1 |  |  |
| HSD11B1 |  |  |
| PLSCR4 |  |  |
| PAFAH1B3 | |  |
| AACS |  |  |
| SPC25 |  |  |
| F8 |  |  |
| HAGH |  |  |
| ANKRD55 | |  |
| IMPDH2 |  |  |
| ABHD6 |  |  |
| RIPK4 |  |  |
| PINK1 |  |  |
| COL4A1 |  |  |
| DSN1 |  |  |
| CDC45 |  |  |
| FEN1 |  |  |
| NSUN6 |  |  |
| ABT1 |  |  |
| CD300A |  |  |
| LMNB1 |  |  |
| SERPINI1 |  |  |
| PLXNC1 |  |  |
| GINS2 |  |  |
| ACADL |  |  |
| DPT |  |  |
| EPHX2 |  |  |
| LSM14B |  |  |
| NUP62 |  |  |
| ADH1C |  |  |
| DGCR2 |  |  |
| ASRGL1 |  |  |
| MNS1 |  |  |
| EHHADH |  |  |
| SLC22A1 |  |  |
| ORC6 |  |  |
| ANO10 |  |  |
| ALAD |  |  |
| SLC27A5 |  |  |
| PFDN4 |  |  |
| SLC10A1 |  |  |
| ICK |  |  |
| PCK2 |  |  |
| NR1I3 |  |  |
| SMC2 |  |  |
| GINS4 |  |  |
| PVALB |  |  |
| SHCBP1 |  |  |
| ABCA6 |  |  |
| LAPTM4B |  |  |
| CIDEB |  |  |
| MT1HL1 |  |  |
| PEMT |  |  |
| BTBD2 |  |  |
| GBA3 |  |  |
| FCGR2B |  |  |
| MCM6 |  |  |
| SC5D |  |  |
| CDA |  |  |
| NCAPG |  |  |
| PKN1 |  |  |
| ZWINT |  |  |
| ACAT1 |  |  |
| MS4A6A |  |  |
| PBLD |  |  |
| C1R |  |  |
| IGHM |  |  |
| AURKA |  |  |
| AGTR1 |  |  |
| FNBP1L |  |  |
| FXYD1 |  |  |
| CDK5 |  |  |
| CD4 |  |  |
| ALDH8A1 |  |  |
| KIF18B |  |  |
| GHR |  |  |
| PON3 |  |  |
| SF3A2 |  |  |
| NRAS |  |  |
| FOSB |  |  |
| ABCA8 |  |  |
| PPIH |  |  |
| PHLDA1 |  |  |
| SYNE1 |  |  |
| GBAP1 |  |  |
| COLEC11 |  |  |
| NSUN5P1 | |  |
| TRAF2 |  |  |
| CCR1 |  |  |
| BHMT |  |  |
| PAMR1 |  |  |
| DCN |  |  |
| TMEM106C | |  |
| PDE2A |  |  |
| CDKN2A |  |  |
| EMC9 |  |  |
| RCL1 |  |  |
| C6 |  |  |
| BUB1 |  |  |
| GNMT |  |  |
| RNF144A |  |  |
| GZMH |  |  |
| FBP1 |  |  |
| HAAO |  |  |
| CYP2C8 |  |  |
| CYP2E1 |  |  |
| HS2ST1 |  |  |
| ECM2 |  |  |
| AKR1D1 |  |  |
| CTH |  |  |
| PLCB1 |  |  |
| DONSON | |  |
| H2AFX |  |  |
| SCUBE3 |  |  |
| VSIG4 |  |  |
| MSH2 |  |  |
| HTRA2 |  |  |
| ZBED8 |  |  |
| ASS1 |  |  |
| EPHA2 |  |  |
| ALAS1 |  |  |
| EHD3 |  |  |
| GPSM2 |  |  |
| ARL4A |  |  |
| CSAD |  |  |
| PPAP2B |  |  |
| COPE |  |  |
| MAPK1 |  |  |
| RAB17 |  |  |
| STIP1 |  |  |
| MSRA |  |  |
| MRC1 |  |  |
| RRM1 |  |  |
| SNRPD1 |  |  |
| FOLH1B |  |  |
| BDH2 |  |  |
| SLC6A12 |  |  |
| SERPINB9 | |  |
| GLYAT |  |  |
| UBE2C |  |  |
| TMPO |  |  |
| MPPED1 |  |  |
| HMGB2 |  |  |
| AZGP1P1 |  |  |
| KNTC1 |  |  |
| KIF11 |  |  |
| ZWILCH |  |  |
| AOC3 |  |  |
| SNRPC |  |  |
| CHST4 |  |  |
| FAM127B |  |  |
| EMR1 |  |  |
| CYP2C9 |  |  |
| ATP1A1 |  |  |
| ALDOA |  |  |
| GCKR |  |  |
| PRUNE |  |  |
| PRIM2 |  |  |
| POLE2 |  |  |
| SCRIB |  |  |
| SLC35E3 |  |  |
| KLHL2 |  |  |
| CBX1 |  |  |
| KAZN |  |  |
| C7 |  |  |
| ZNF74 |  |  |
| MT1M |  |  |
| CUL9 |  |  |
| DAK |  |  |
| SULT1C2 |  |  |
| CCT6A |  |  |
| ACAA2 |  |  |
| ATAD2 |  |  |
| NUDT1 |  |  |
| ITGB1BP1 |  |  |
| SLC16A4 |  |  |
| PDIA6 |  |  |
| RDH16 |  |  |
| LY6E |  |  |
| CHEK1 |  |  |
| ZG16 |  |  |
| CAT |  |  |
| CD160 |  |  |
| GCDH |  |  |
| ADH6 |  |  |
| HJURP |  |  |
| CKAP4 |  |  |
| TYMS |  |  |
| KMO |  |  |
| CBX8 |  |  |
| GNE |  |  |
| LRRC42 |  |  |
| IGF2BP3 |  |  |
| DLG5 |  |  |
| ACOX1 |  |  |
| SOCS2 |  |  |
| GCGR |  |  |
| KLRB1 |  |  |
| IGFBP3 |  |  |
| MARK4 |  |  |
| GRAMD1C | |  |
| SLC2A8 |  |  |
| FABP5 |  |  |
| CD8A |  |  |
| FOS |  |  |
| MT1E |  |  |
| USP39 |  |  |
| GYS2 |  |  |
| TACC3 |  |  |
| DTYMK |  |  |
| CLGN |  |  |
| DNAJC6 |  |  |
| CDKN2C |  |  |
| IRAK1 |  |  |
| MPC1 |  |  |
| MCM2 |  |  |
| LIFR |  |  |
| MS4A5 |  |  |
| LPA |  |  |
| STEAP3 |  |  |
| COL15A1 |  |  |
| SCAND2P | |  |
| SLCO1B3 |  |  |
| AFM |  |  |
| RND3 |  |  |
| ALDH2 |  |  |
| CYP3A4 |  |  |
| RCN2 |  |  |
| CD1D |  |  |
| GADD45G | |  |
| MAT1A |  |  |
| GABARAPL1 | |  |
| MT1X |  |  |
| SMC4 |  |  |
| NGFR |  |  |
| KLRF1 |  |  |
| KIF23 |  |  |
| MT2A |  |  |
| CYP1A1 |  |  |
| CBR4 |  |  |
| GPC3 |  |  |
| RAN |  |  |
| RCAN1 |  |  |
| ADH1A |  |  |
| AXL |  |  |
| N4BP2L1 |  |  |
| CHCHD3 |  |  |
| UBE2S |  |  |
| LOX |  |  |
| INTS8 |  |  |
| PROC |  |  |
| NMRK1 |  |  |
| GNA14 |  |  |
| SERPINA4 | |  |
| ECHS1 |  |  |
| ZGPAT |  |  |
| DLGAP5 |  |  |
| TTK |  |  |
| KIAA0101 |  |  |
| PARPBP |  |  |
| SSR2 |  |  |
| SHARPIN |  |  |
| BUB1B |  |  |
| POLR2K |  |  |
| GINS1 |  |  |
| CENPF |  |  |
| PXDC1 |  |  |
| CDK4 |  |  |
| HELLS |  |  |
| MTHFD1 |  |  |
| ACLY |  |  |
| CDC6 |  |  |
| PRCC |  |  |
| DCXR |  |  |
| NPY1R |  |  |
| MDK |  |  |
| DTL |  |  |
| OIP5 |  |  |
| FAM134B |  |  |
| LCAT |  |  |
| C1orf112 |  |  |
| RALY |  |  |
| ETFDH |  |  |
| GPD1 |  |  |
| RRM2 |  |  |
| WSB2 |  |  |
| NDRG2 |  |  |
| KCNN2 |  |  |
| CD14 |  |  |
| PBK |  |  |
| ACSM5 |  |  |
| HMGCL |  |  |
| HMGA1 |  |  |
| MAN1C1 |  |  |
| ACSL4 |  |  |
| TUBG1 |  |  |
| CNIH4 |  |  |
| MCM3 |  |  |
| ST3GAL6 |  |  |
| RFC4 |  |  |
| SMYD3 |  |  |
| TPX2 |  |  |
| CXCL12 |  |  |
| CEP55 |  |  |
| GPM6A |  |  |
| GSTZ1 |  |  |
| ZNF706 |  |  |
| SRPX |  |  |
| LILRB5 |  |  |
| ABAT |  |  |
| EXPH5 |  |  |
| APOF |  |  |
| FANCG |  |  |
| KPNA2 |  |  |
| CENPM |  |  |
| C1QTNF1 | |  |
| CXCL14 |  |  |
| ECT2 |  |  |
| E2F8 |  |  |
| H2AFZ |  |  |
| ESM1 |  |  |
| CDKN3 |  |  |
| CYP2A6 |  |  |
| CDC20 |  |  |
| MYBL2 |  |  |
| STARD5 |  |  |
| HBB |  |  |
| MT1H |  |  |
| PTTG1 |  |  |
| MT1G |  |  |
| FYN |  |  |
| SPDL1 |  |  |
| SRD5A2 |  |  |
| ADD2 |  |  |
| KLKB1 |  |  |
| GMNN |  |  |
| MCM4 |  |  |
| CCNB2 |  |  |
| EZH2 |  |  |
| CCNB1 |  |  |
| CDHR2 |  |  |
| KIF14 |  |  |
| SORL1 |  |  |
| SPP2 |  |  |
| KIF4A |  |  |
| CRHBP |  |  |
| UBE2M |  |  |
| MASP2 |  |  |
| MT1F |  |  |
| MAD2L1 |  |  |
| ACAA1 |  |  |
| CYP2A7 |  |  |
| SERPINH1 | |  |
| PLVAP |  |  |
| CYP26A1 |  |  |
| ILF2 |  |  |
| NDC80 |  |  |
| ECM1 |  |  |
| STAB2 |  |  |
| CETP |  |  |
| SPINK1 |  |  |
| ASPM |  |  |
| CYP2B6 |  |  |
| HN1 |  |  |
| HAMP |  |  |
| HSPB1 |  |  |
| ADRA1A |  |  |
| CYP39A1 |  |  |
| CDCA3 |  |  |
| RACGAP1 | |  |
| PRC1 |  |  |
| CDH19 |  |  |
| LYVE1 |  |  |
| COLEC10 |  |  |
| PLAC8 |  |  |
| IGFALS |  |  |
| FCN2 |  |  |
| PTH1R |  |  |
| DBH |  |  |
| SAC3D1 |  |  |
| CDK1 |  |  |
| CPEB3 |  |  |
| DNASE1L3 | |  |
| F11 |  |  |
| FEZ1 |  |  |
| MELK |  |  |
| FCN3 |  |  |
| TOP2A |  |  |
| CDC37L1 |  |  |
| NUSAP1 |  |  |
| ENAH |  |  |
| CD5L |  |  |
| ESR1 |  |  |
| CYP1A2 |  |  |
| NAT2 |  |  |
| KIF20A |  |  |
| VIPR1 |  |  |
| MARCO |  |  |
| CFP |  |  |
| CLEC1B |  |  |
| FOXM1 |  |  |
| CLEC4M |  |  |
| FAM189B |  |  |
