# Supplementary material for: Exploring the mechanisms underlying the therapeutic effect of the Radix Bupleuri-Rhizoma Cyperi herb pair on hepatocellular carcinoma using multilevel data integration and molecular docking
Source: Aging (Albany NY). 2022 Nov 18;14(22):9103–27. doi: 10.18632/aging.204388 (PMC9740357; doi:10.18632/aging.204388)
Supplement: Supplementary Tables 2-4 and 6-8 [file aging-14-204388-s002.pdf]

SUPPLEMENTARY TABLES

Supplementary Table 2. Degree rank of nodes in the PPI network of 224 DEGs in HCC (Node_degree >0, Blanks are represented as none).

Symbol	DEGREE	MCODE_CLUSTER_ID	MCODE_TYPE	Symbol	DEGREE	MCODE_CLUSTER_ID	MCODE_TYPE
CDK1	27	0		PBK	3	0	
CYP2E1	19	1	Clustered	IGF2BP3	3	0	
KIF20A	17	0		TNFSF14	3	0	
PRC1	15	0		SPP1	3	0	
CYP26A1	14	1	Clustered	SHBG	3	0	
CYP3A4	14	1	Clustered	PDGFRA	3	0	
CYP2C9	14	1	Clustered	MT1G	3	0	
CYP2B6	14	1	Clustered	MEP1A	3	0	
CYP2A6	14	1	Seed	ITIH4	3	0	
PLG	13	4	Clustered	GYS2	3	0	
ESR1	13	0		GPC3	3	0	
CYP1A2	13	1	Clustered	GCGR	3	0	
CYP1A1	13	1	Clustered	DCN	3	0	
KIF23	12	8	Clustered	C9	3	6	Clustered
CYP4A11	12	1	Clustered	C8A	3	6	Seed
CYP2C8	12	1	Clustered	BHMT	3	0	
CCNB1	11	0		BARD1	3	0	
CYP3A7-CYP3A51P	10	1	Clustered	NAT2	3	0	
CYP39A1	10	1	Clustered	ZGPAT	2	0	
NUP62	10	0		CFHR5	2	0	
RUVBL2	10	0		SMYD3	2	0	
NDC80	10	0		CFHR4	2	0	
TOP2A	10	0		FAM189B	2	0	
MAD2L1	9	0		VNN1	2	0	
ACSL4	9	8	Clustered	TFPI2	2	0	
PTGS2	8	0		VIPR1	2	0	
PKM	8	0		SULT1C2	2	0	
MMP1	8	4	Clustered	SRD5A2	2	0	
IGF1	8	4	Clustered	SLC22A1	2	0	
TPX2	7	0		CCL19	2	0	
CCL2	7	0		S100P	2	0	
OTC	7	8	Clustered	RELN	2	0	
FOS	7	0		PCK1	2	0	
CYP27A1	7	0		NPY1R	2	0	
CYP2A7	7	0		MT1M	2	0	
CPS1	7	8	Seed	HRG	2	0	
CDC20	7	0		HBB	2	0	
TTK	6	0		GSTZ1	2	0	
PON1	6	3	Clustered	FMO3	2	0	
MBL2	6	2	Clustered	CLGN	2	0	

MAT1A	6	0		BCHE	2	0
HSD11B1	6	0		ADRA1A	2	0
FCN2	6	2	Clustered	SHARPIN	1	0
ADH1B	6	5	Clustered	KDM8	1	0
COLEC11	5	2	Clustered	GBA3	1	0
AKR1B10	5	0		INAVA	1	0
ZWILCH	5	0		C1orf112	1	0
RACGAP1	5	0		STAB2	1	0
SDS	5	0		P2RY13	1	0
MASP2	5	2	Clustered	DCXR	1	0
COLEC10	5	2	Seed	SAC3D1	1	0
FCN3	5	2	Clustered	NPC1L1	1	0
CDC45	5	7	Clustered	CDH19	1	0
AKR1D1	5	0		SLCO1B3	1	0
NOTCH1	5	0		GNMT	1	0
MME	5	0		GLS2	1	0
LCAT	5	3	Clustered	ABCA6	1	0
KLKB1	5	0		CPEB3	1	0
IGFBP3	5	4	Clustered	SLC27A5	1	0
HMGA1	5	0		LYVE1	1	0
HELLS	5	0		SLCO1B1	1	0
FOXM1	5	0		TACC3	1	0
FBP1	5	0		ABCA8	1	0
FANCG	5	0		CLEC4M	1	0
CTH	5	0		GAL3ST1	1	0
C7	5	6	Clustered	MARCO	1	0
ADH6	5	5	Clustered	RDH16	1	0
ADH1C	5	5	Clustered	SPP2	1	0
ADH1A	5	5	Seed	SLC10A1	1	0
ASPM	4	0		SERPINI1	1	0
CENPM	4	0		PAFAH1B3	1	0
MCM10	4	7	Clustered	NGFR	1	0
HJURP	4	0		MT1F	1	0
CEP55	4	0		MT1E	1	0
SPDL1	4	0		LUM	1	0
GINS2	4	7	Seed	IGLC1	1	0
GMNN	4	0		IGHM	1	0
GINS1	4	7	Clustered	IFIT1	1	0
MELK	4	0		HPD	1	0
CFP	4	0		HGFAC	1	0
MYBL2	4	0		GPM6A	1	0
LPA	4	3	Clustered	GPD1	1	0
IL13RA2	4	0		DPT	1	0
IGFALS	4	4	Seed	DBH	1	0
FOSB	4	0		COL15A1	1	0
CETP	4	3	Seed	CHI3L1	1	0
SERPINH1	4	0		CD14	1	0

C6	4	6	Clustered	ACADL	1	0
APOF	4	3	Clustered			

Supplementary Table 3. MCODE components.

Network	Annotation
MyList	R-HSA-211945|Phase I - Functionalization of compounds|-22.4;R-HSA-211859|Biological oxidations|-21.4;GO:0008202|steroid metabolic process|-21.3
MyList_MCODE_ALL	R-HSA-211945|Phase I - Functionalization of compounds|-28.2;WP206|Fatty acid omega-oxidation|-25.4;hsa00830|Retinol metabolism|-24.3
MyList_SUB1_MCODE_1	R-HSA-211897|Cytochrome P450 - arranged by substrate type|-32.4;R-HSA-211945|Phase I - Functionalization of compounds|-29.7;WP43|Oxidation by cytochrome P450|-28.8
MyList_SUB1_MCODE_2	R-HSA-166662|Lectin pathway of complement activation|-22.6;GO:0001867|complement activation, lectin pathway|-21.7;R-HSA-166786|Creation of C4 and C2 activators|-20.5
MyList_SUB1_MCODE_3	GO:0008203|cholesterol metabolic process|-9.0;GO:1902652|secondary alcohol metabolic process|-8.8;GO:0016125|sterol metabolic process|-8.7
MyList_SUB1_MCODE_4	R-HSA-381426|Regulation of Insulin-like Growth Factor (IGF) transport and uptake by Insulin-like Growth Factor Binding Proteins (IGFBPs)|-12.0;CORUM:541|IGF1-IGFBP3-ALS complex|-11.7;hsa04935|Growth hormone synthesis, secretion and action|-6.2
MyList_SUB1_MCODE_5	GO:0006069|ethanol oxidation|-14.4;R-HSA-71384|Ethanol oxidation|-13.8;WP206|Fatty acid omega-oxidation|-13.4
MyList_SUB1_MCODE_6	R-HSA-166665|Terminal pathway of complement|-14.7;GO:0019835|cytolysis|-12.8;WP545|Complement activation|-12.7
MyList_SUB1_MCODE_7	R-HSA-176974|Unwinding of DNA|-9.7;GO:0006261|DNA-templated DNA replication|-9.4;GO:0006268|DNA unwinding involved in DNA replication|-8.9
MyList_SUB1_MCODE_8	GO:0043604|amide biosynthetic process|-4.7;GO:0043603|cellular amide metabolic process|-4.2

Pathway and process enrichment analysis has been independently applied to each MCODE component, and the three best-scoring terms by *P*-values were retained as functional descriptions of the corresponding components (Top three best *P*-value terms were retained).

Supplementary Table 4. A total of 31 CXP-active compounds were identified in TCMSP based on two criteria of DL ≥0.18 and OB ≥30%.

MOL ID	Molecule name	OB (%)	DL	Herb
MOL000098	Quercetin	46.43	0.28	*Radix Bupleuri/Rhizoma Cyperi*
MOL000449	Stigmasterol	43.83	0.76	*Radix Bupleuri/Rhizoma Cyperi*
MOL000354	Isorhamnetin	49.6	0.31	*Radix Bupleuri/Rhizoma Cyperi*
MOL000422	Kaempferol	41.88	0.24	*Radix Bupleuri/Rhizoma Cyperi*
MOL004598	3,5,6,7-tetramethoxy-2-(3,4,5-trimethoxyphenyl) chromone	31.97	0.59	*Radix Bupleuri*
MOL001645	Linoleyl acetate	42.1	0.2	*Radix Bupleuri*
MOL004609	Areapillin	48.96	0.41	*Radix Bupleuri*
MOL013187	Cubebin	57.13	0.64	*Radix Bupleuri*
MOL004624	Longikaurin A	47.72	0.53	*Radix Bupleuri*
MOL004644	Sainfuran	79.91	0.23	*Radix Bupleuri*

MOL004648	Troxerutin	31.6	0.28	*Radix Bupleuri*
MOL004653	(+)-Anomalin	46.06	0.66	*Radix Bupleuri*
MOL004702	Saikosaponin c_qt	30.5	0.63	*Radix Bupleuri*
MOL004718	α-Spinasterol	42.98	0.76	*Radix Bupleuri*
MOL000490	Petunidin	30.05	0.31	*Radix Bupleuri*
MOL002776	Baicalin	40.12	0.75	*Radix Bupleuri*
MOL004628	Octalupine	47.82	0.28	*Radix Bupleuri*
MOL003542	8-Isopentenyl-kaempferol	38.04	0.39	*Rhizoma Cyperi*
MOL000358	*β*-sitosterol	36.91	0.75	*Rhizoma Cyperi*
MOL000359	Sitosterol	36.91	0.75	*Rhizoma Cyperi*
MOL004027	1,4-Epoxy-16-hydroxyheneicos-1,3,12,14,18-pentaene	45.1	0.24	*Rhizoma Cyperi*
MOL004053	Isodalbergin	35.45	0.2	*Rhizoma Cyperi*
MOL004058	Khellin	33.19	0.19	*Rhizoma Cyperi*
MOL004059	Khellol glucoside	74.96	0.72	*Rhizoma Cyperi*
MOL003044	Chryseriol	35.85	0.27	*Rhizoma Cyperi*
MOL004068	Rosenonolactone	79.84	0.37	*Rhizoma Cyperi*
MOL004071	Hyndarin	73.94	0.64	*Rhizoma Cyperi*
MOL004074	Stigmasterol glucoside_qt	43.83	0.76	*Rhizoma Cyperi*
MOL004077	Sugeonyl acetate	45.08	0.2	*Rhizoma Cyperi*
MOL000006	Luteolin	36.16	0.25	*Rhizoma Cyperi*
MOL010489	Resivit	30.84	0.27	*Rhizoma Cyperi*

Supplementary Table 6. MCODE components.

Network	Annotation
MyList	WP2882|Nuclear receptors meta-pathway|-18.5;GO:0009410|response to xenobiotic stimulus|-15.4;GO:0046686|response to cadmium ion|-14.5
MyList_MCODE_ALL	hsa05207|Chemical carcinogenesis - receptor activation|-15.1;WP2882|Nuclear receptors meta-pathway|-13.6;GO:0009410|response to xenobiotic stimulus|-12.8
MyList_SUB1_MCODE_1	M176|PID FOXM1 PATHWAY|-13.7;WP2431|Spinal cord injury|-11.3;hsa05224|Breast cancer|-10.8
MyList_SUB1_MCODE_2	WP697|Estrogen metabolism|-12.3;GO:0001676|long-chain fatty acid metabolic process|-12.2;GO:0006633|fatty acid biosynthetic process|-12.1

Pathway and process enrichment analysis has been independently applied to each MCODE component, and the three best-scoring terms by *P*-values were retained as functional descriptions of the corresponding components (Top three best *P*-value terms were retained).

Supplementary Table 7. Degree rank of nodes in the PPI network of 44 potential therapeutic targets for CXP in HCC (Blanks are represented as none).

Symbol	DEGREE	MCODE_CLUSTER_ID	MCODE_TYPE
MYC	14	1	Clustered
CDK1	13	1	Clustered
FOS	11	1	Clustered
MAPK1	10	1	Clustered
ESR1	9	1	Clustered
AR	7	0	
CDK4	7	1	Seed
CYP1A2	5	2	Clustered
CCL2	5	0	
CYP1A1	4	2	Clustered
CYP3A4	4	2	Clustered
GSTM1	4	2	Clustered
HMOX1	4	0	
HSPB1	4	0	
MMP1	4	0	
TOP2A	4	0	
CAT	3	0	
CCNB1	3	0	
CDKN2A	3	0	
CHEK1	3	0	
PTGS2	3	0	
AKR1C3	3	2	Seed
HK2	2	0	
IGFBP3	2	0	
SPP1	2	0	
MGAM	2	0	
NR1I3	2	0	
ACAA2	2	0	
ACACA	1	0	
ADH1C	1	0	
CA2	1	0	
NQO1	1	0	
PON1	1	0	
NR1I2	1	0	
NCF1	1	0	

Supplementary Table 8. Binding energies of different CXP active components binding to cell cycle-related proteins based on molecular docking analysis.

Protein (PDB ID)	Molecule name	Binding Energy (kcal/mol)	Herb
CCNB1 (4Y72)	Luteolin	−6.7	*Rhizoma Cyperi*
CCNB1(4Y72)	Quercetin	−9.1	*Radix Bupleuri/Rhizoma Cyperi*
CDK4 (2W9Z)	Luteolin	−8.6	*Rhizoma Cyperi*
CDKN2A (1DC2)	Quercetin	−6.8	*Radix Bupleuri/Rhizoma Cyperi*
CHEK1 (2HOG)	8-Isopentenyl-kaempferol	−9.3	*Rhizoma Cyperi*
CHEK1 (2HOG)	Areapillin	−7.6	*Radix Bupleuri*
CHEK1 (2HOG)	β-sitosterol	−8.8	Rhizoma Cyperi
CHEK1 (2HOG)	Chryseriol	−8.3	*Rhizoma Cyperi*
CHEK1 (2HOG)	Cubebin	−9	*Radix Bupleuri*
CHEK1 (2HOG)	Hyndarin	−9	*Rhizoma Cyperi*
CHEK1 (2HOG)	Isorhamnetin	−8.5	*Radix Bupleuri/Rhizoma Cyperi*
CHEK1 (2HOG)	Kaempferol	−8.5	*Radix Bupleuri/Rhizoma Cyperi*
CHEK1 (2HOG)	Khellin	−7.3	*Rhizoma Cyperi*
CHEK1 (2HOG)	Quercetin	−8.8	*Radix Bupleuri/Rhizoma Cyperi*
